# Assessment and Error Analysis of Terra‐MODIS and MISR Cloud‐Top Heights Through Comparison With ISS‐CATS Lidar

**DOI:** 10.1029/2020JD034281

**Published:** 2021-05-02

**Authors:** Arka Mitra, Larry Di Girolamo, Yulan Hong, Yizhe Zhan, Kevin J. Mueller

**Affiliations:** ^1^ University of Illinois Urbana‐Champaign IL USA; ^2^ Metservice Ltd. Wellington New Zealand; ^3^ Jet Propulsion Laboratory California Institute of Technology Pasadena CA USA

**Keywords:** cloud‐top heights, error analysis, MISR, stereo‐opacity bias, Terra MODIS, Terra satellite

## Abstract

Cloud‐top heights (CTH) from the Multiangle Imaging Spectroradiometer (MISR) and the Moderate Resolution Imaging Spectroradiometer (MODIS) on Terra constitute our longest‐running single‐platform CTH record from a stable orbit. Here, we provide the first evaluation of the Terra Level 2 CTH record against collocated International Space Station Cloud‐Aerosol Transport System (CATS) lidar observations between 50ºN and 50ºS. Bias and precision of Terra CTH relative to CATS is shown to be strongly tied to cloud horizontal and vertical heterogeneity and altitude. For single‐layered, unbroken, optically thick clouds observed over all altitudes, the uncertainties in MODIS and MISR CTH are −540 ± 690 m and −280 ± 370 m, respectively. The uncertainties are generally smaller for lower altitude clouds and larger for optically thin clouds. For multi‐layered clouds, errors are summarized herein using both absolute CTH and CATS‐layer‐altitude proximity to Terra CTH. We show that MISR detects the lower cloud in a two‐layered system, provided top‐layer optical depth <∼0.3, but MISR low‐cloud CTH errors are unaltered by the presence of thin cirrus. Systematic and random errors are propagated to explain inter‐sensor disagreements, as well as to provide the first estimate of the MISR stereo‐opacity bias. For MISR, altitude‐dependent wind‐retrieval bias (−90 to −110 m) and stereo‐opacity bias (−60 to −260 m) and for MODIS, CO_2_‐slicing bias due to geometrically thick cirrus leads to overall negative CTH bias. MISR’s precision is largely driven by precision in retrieved wind‐speed (3.7 m s^−1^), whereas MODIS precision is driven by forward‐modeling uncertainty.

## Key Points

- We present the first semi‐global (50°N–50°S) comparison of Terra cloud‐top heights with coincident samples from a space‐based lidar
- Using lidar as truth, Terra cloud‐top height bias and precision are summarized as a function of cloud geometrical and optical properties
- With the first measurement of stereo‐opacity bias (−60 to −260 m, depending on cloud type), MISR cloud height error‐budget is closed

## 1 Introduction

Cloud altitude feedback is an important component of cloud feedbacks (Zelinka et al., 2017), with inter‐model differences in cloud feedbacks being the largest source of uncertainty in climate predictions (Boucher et al., 2013; Dufresne & Bony, 2008). One of the suggested techniques to lower these inter‐model differences is to compare short‐term model predictions with accurate global trends in cloud vertical distribution from stable satellite‐based sensors. However, short‐term trends in cloud‐top height (CTH) are often quite small in magnitude and dominated by natural variability in the ocean‐atmosphere system (Davies et al., 2017; Geiss & Marchand, 2019). Ohring et al. (2004) recommended a CTH accuracy of 150 m and a stability of 30 m/decade from a satellite sensor for monitoring decadal changes in CTH. Accurate CTH is also necessary in other meteorological research, such as in predicting vertical variations of freezing layers (Van Diedenhoven et al., 2016). As a result, it is imperative that the error characteristics of public standard CTH products be fully established and understood

CTH retrievals are broadly classified as active or passive. Popular passive CTH retrieval techniques include CO_2_‐slicing (Menzel et al., 1983), 11‐μm brightness temperature (Menzel et al., 2008), and simultaneous retrieval of CTH and winds through stereo photogrammetry (Muller et al., 2002). These passive techniques rely on a single‐layered cloud assumption for a given field‐of‐view—an assumption that is valid only ∼75%–80% of the time over the globe (Stubenrauch et al., 2013). Active sensors (radars and lidars) can provide detailed hydrometeor vertical distributions (Hagihara et al., 2010; Li et al., 2015), unlike passive sensors. As a result, many previous studies (Marchand et al., 2007; Naud et al., 2002, 2004, 2007) have employed active sensor CTH as the truth to quantify passive sensor CTH errors. Those studies arrived at the consensus that multi‐layered clouds can lead to large differences in retrieved CTH among passive sensors. In those studies, and the present one, CTHs from two imagers onboard the Terra satellite—the Multiangle Imaging Spectroradiometer (MISR) and the Moderate Resolution Imaging Spectroradiometer (MODIS—were analyzed. Terra has provided us with a consistent equator crossing time or equatorial crossing time (ECT) (Stubenrauch et al., 2013; Zhao et al., 2016) for nearly two decades and is our longest‐running stable climate record of CTH. Hence, understanding the CTH error characteristics in retrievals from MISR and MODIS radiances is essential for interpreting CTH variability within their records to shed light on their strengths and weaknesses, to combine their strengths for improved CTH characterization, and to better inform future satellite missions on system design choices for reducing uncertainty in CTH retrievals.

Collection five 1‐ and 5‐km resolution CTHs from the MODIS instrument on‐board Aqua were compared against near‐coincident CALIPSO CTHs globally for 2 months of 2006/2007 by Holz et al. (2008); made possible because both these instruments are part of NASA’s A‐Train constellation of satellites (1:30 pm ECT). Holz et al. (2008) reported globally averaged CTH differences between the 1‐km MODIS and CALIPSO CTH to be −1.4 ± 2.9 km (the 5‐km product exhibited worse accuracy and precision due to poorer resolution). Through a detailed analysis, the high negative bias of CTH was found to be largely due to the presence of optically thin high clouds (often, in multi‐layered situations), and a failure of the CO_2_‐slicing algorithm to converge to a solution in many high cloud scenes (in which case the less precise 11 μm brightness temperature technique would be applied). Random errors, meanwhile, were attributed to incorrect lapse‐rates for marine low‐level clouds and application of the brightness temperature technique for high clouds (Section 4.5.2 provides an in‐depth discussion of these errors). The last two issues were specifically addressed in a series of improvements (Baum et al., 2012) that resulted in the latest Collection six MODIS 1‐km CTH product. Comparisons of Aqua MODIS Collection six non‐polar CTH against CALIPSO CTH showed higher application of CO_2_‐slicing than in Collection five for single‐layered cirrus (less miscasting of high clouds as low clouds) and a reduction of the low‐cloud positive bias from 424 m in Collection five to 197 m in Collection six (Baum et al., 2012). While ostensibly the same, Terra MODIS and Aqua MODIS are subject to key differences for CTH determination that stem from diurnal variability of lapse rates and cloud characteristics between morning and afternoon (Eastman & Warren, 2014), as well as the absence of Band 34 (13.6 µm) on Terra due to high noise. As such, an independent study of Terra MODIS is also necessary, as well as for validating the Collection six CTH product.

MISR employs a stereoscopic technique for determining CTH and cloud‐top advection or “winds,” simultaneously (Mueller et al., 2013; Muller et al., 2002). The original CTH product, referenced to as TC_STEREO, often reported an uncertainty of 562 m, but this was specific to the error made if stereo correspondence was off by a single pixel (Moroney et al., 2002). Validation against ground‐based radars and lidars showed that CTH uncertainties tended to be less than 1 km, irrespective of cloud height (Marchand et al., 2007; Naud et al., 2004). These studies showed that, when an optically thin upper cloud overlies an optically thick lower cloud (which is often the case in multi‐layered situations), MISR returns the CTH of the lower cloud, provided the upper cloud optical depth is less than 0.3–0.5, depending on surface type (Marchand et al., 2007). This is because lower cloud layers often provide the greatest observed spatial contrast in MISR’s visible to near‐IR images, even in the presence of thin upper clouds. TC_STEREO also produced many no‐retrievals, in part due to overly strict quality control. More recently, the MISR algorithm underwent a series of improvements (Horváth, 2013) to generate the latest stereo product, called TC_CLOUD (Mueller et al., 2013). Although direct validation of TC_CLOUD CTH against active sensors has not yet been done, Horváth (2013) and Mueller et al. (2017) compared MISR winds with geostationary IR atmospheric motion‐vectors (AMVs) from Meteosat‐9 and GOES, respectively, revealing a pattern of mean and root mean‐squared (RMS) differences between MISR and geostationary wind heights that vary with altitude and location (Section 4.5.1 provides an in‐depth discussion of these errors). Averaged globally, wind‐related CTH bias relative to IR AMV heights were found to be ∼ −200 m, with associated precision ranging from 0.5 to 1.0 km, depending on the dataset. The large deviation in the random error estimates can be attributed to the inherent uncertainties of the IR AMV retrievals; however, better estimates require precise cloud height measurements, such as from a lidar.

The lack of a space‐based active sensor with sufficient orbital overlap with Terra has so far impeded a global validation of MISR and Terra‐MODIS CTHs. To realize our goal of validating Level 2 Terra CTH, the database of “true” active‐sensor CTH is taken from the ISS‐CATS (Yorks, Mcgill, et al., 2016a). ISS‐CATS or simply, CATS (Cloud‐ Aerosol Transport System) was a space‐based lidar that operated from the Japanese Experiment Module‐Exposed Facility of the International Space Station (ISS) from 2015 through 2017. Although too short‐lived to be a climate record, CATS was uniquely suited for a quasi‐global validation of CTH from Terra‐based sensors. Here, we use the CATS dataset to examine the error characteristics of MISR and Terra‐MODIS CTHs.

Section 2 briefly describes the instruments, their orbits, and the data sources. Section 3 elucidates the collocation among CATS, MISR, and MODIS pixels and quantifies the random errors within our methods. Section 4 delves into CTH differences from the inter‐comparison of the three instruments, the global distribution of these differences, and the chief reasons behind the disagreements. Concluding remarks follow in Section 5.

## 2 Data and Instruments

The flagship of NASA’s Earth Observing System (EOS), Terra, is a near‐polar, sun‐synchronous satellite orbiting the Earth at a nominal altitude of 705 km above the surface, making its equator overpasses at 10:30 a.m. local time. MISR and MODIS are two instruments on Terra that use two completely independent techniques for retrieving CTH. MISR employs a stereoscopic technique using 0.67‐μm (“Red” channel), 275‐m resolution radiance from the three least oblique angles (nadir and ±26.1°) to estimate CTH (Muller et al., 2002). One advantage of a stereoscopic technique over other passive CTH retrievals is that a stereo CTH is not sensitive to radiometric calibration (Naud et al., 2002). The operational MISR algorithm first estimates cloud‐top winds and then stereo heights for each 1.1‐km pixel in a scene. The MISR data used here are the Level 2 TC_CLOUD Version F01_0001 orbit‐level product, which provides a 1.1‐km “wind‐corrected” CTH over a swath of width 380 km.

MODIS is a broad‐swath (swath width ∼2,330 km) imager with 36 spectral channels that has a nadir spatial resolution ranging from 250 to 1,000 m, depending on the spectral channel. The MISR swath lies completely within the MODIS swath. MODIS employs a CO_2_‐slicing technique (Menzel et al., 2008) for CTH estimation, designed to calculate the cloud‐top pressure (CTP) and effective cloud amount for geometrically thin, single‐layered mid‐level and high clouds. These quantities are derived from ratios of differences between cloudy and clear‐sky radiances from any of the following pairs: 14.2/13.9 μm, 13.9/13.6 μm, 13.9/13.3 μm, or 13.6/13.3 μm, with MODIS CTP reporting the solution of the highest wavelength band‐pair whose radiance difference exceeds instrument noise in the individual bands. It is assumed that cloud emissivity is equal for both wavelengths in the pair, an assumption better suited for ice clouds than water clouds. CTP retrieval occurs at 1‐km resolution, provided that at least 4 out of the 25 pixels in a 5 × 5 pixel window surrounding it were flagged as either cloudy or probably cloudy by the MODIS cloud mask and an independent pixel‐level phase detection flagged ice. CTP is converted to CTH using Global Data Assimilation System (GDAS) model output. For low‐level (CTP > 650 hPa) or liquid‐phase clouds or when none of the band pairs converge to a solution, the 11‐μm brightness temperature (IR BT) technique estimates a cloud‐top temperature (CTT) and from that, a CTP/CTH is calculated from gridded model output, with provisions to adjust the lapse rate for marine stratus (Baum et al., 2012). The Terra MODIS CTH product used here is the Collection 6.1 Level 2 MOD06, which is provided in granule form at a 5‐min temporal resolution.

The ISS is at a mean altitude of 409 km above the Earth, revolving in a nearly circular low‐earth orbit with an inclination of 51.64° and completing about 16 revolutions/day. The Cloud‐Aerosol Transport System (CATS) (McGill et al., 2015; Yorks, Mcgill, et al., 2016a) instrument onboard the ISS operated from February 10, 2015 to October 30, 2017 and consisted of two elastic backscatter lasers that used a combination of low energy, high repetition rate 532‐ and 1,064‐nm pulses (with a footprint of 14.38‐m diameter) to achieve greater output power than any previous space lidar (Pauly et al., 2019). Although instrument failure prevented its multiple intended operating modes, nadir‐only information was retained. During its run, CATS data were continuously downlinked at 60‐m vertical and 350‐m horizontal resolution (except for loss‐of‐signal periods), and then pre‐processed, geo‐located and calibrated to produce CATS Level 1 attenuated total backscatter and depolarization ratio profiles (Yorks, Mcgill, et al., 2016a). Geophysical parameters derived from Level 1 information were compiled into 5‐km resolution Level 2 data (∼14,350‐m Level 1 profiles are processed to yield a 5‐km CATS datum, with the tagged geolocation representing the center of the 5‐km stretch), including depolarization ratio and attenuated backscatter, along with their layer‐integrated values. The CATS layer‐detection algorithm follows the Cloud‐Aerosol Lidar with Orthogonal Polarization (CALIOP) algorithm (Vaughan et al., 2005; Yorks, Mcgill, et al., 2016a), with the main difference being that CATS applied threshold‐based feature‐detection on 5‐km backscatter profiles at 1,064 nm, as opposed to 532 nm for CALIOP. CATS layer‐detection also operated only at a single 5‐km horizontal resolution (60 m vertical resolution), whereas the CALIOP algorithm successively runs at fine to coarse horizontal resolutions ranging from 5 to 80 km in order to detect progressively tenuous layers (Vaughan et al., 2009). Cloud‐aerosol feature‐mask discrimination and cloud phase detection are identical to CALIOP. Details of these techniques can be found in the CATS Algorithm Theoretical Basis Document (Yorks, Palm, et al., 2016). The Level 1 1,064‐nm backscatter and depolarization used for the detection of cloud layers have been validated by comparing against Cloud Physics Lidar (CPL) observations (Yorks, Mcgill, et al., 2016a). CATS Version 2.01 Level 2 Product used in this study provided values at every lidar range‐gate associated with successful layer‐discrimination. For this study, only range‐gates with cloudy feature‐masks were considered. Since our data processing began, CATS Level 2 products have been upgraded to Version 3.01. A comparison of CTHs between the two versions revealed a zero mean difference and a standard deviation of 15 m. This small effect is not surprising since the algorithmic improvements in the version change mainly pertained to aerosol layer detection (see CATS L2O Profile Products Quality Statements: Version 3.00, available online at https://cats.gsfc.nasa.gov/media/docs/CATS_QS_L2O_Profile_3.00.pdf). While we use the lidar as the reference truth for CTH, lidars also suffer from layer detection threshold issues (e.g., Vaughan et al., 2009). We provide an accounting of this uncertainty in the error budgets reported in Section 4.5.1 under a reasonable best‐case scenario, which can be updated for better quantification of the uncertainties in lidar cloud layer heights arise.

## 3 Collocation Methodology

For an accurate inter‐comparison between instruments, one needs to be able to compare spatially and temporally concurrent observations, due to the transient nature of atmospheric conditions. In our case, MODIS has the widest swath, and CTH is stored in 5‐min granules at 1‐km resolution, whereas MISR, with a much narrower swath nestled within the MODIS swath, provides CTH at 1.1‐km resolution that are stored per orbit. This enables a one‐to‐one collocation between MODIS and MISR pixels. However, CATS has a narrow Ground Instantaneous Field‐of‐View (GIFOV) of 14.38‐m diameter, with each successive GIFOV separated by 350 m (i.e., the horizontal resolution of a Level 1 datum). Since CATS does not scan cross‐track, its swath‐width, thus, equals 14.38 m. Each CATS Level 2 datum has an along‐track resolution of 5 km. Thus, when overlap of the Terra and ISS orbits did happen, it was possible to have multiple MODIS and MISR pixels neighboring a single CATS Level 2 point. Here, we choose a one‐to‐one collocation between each CATS point and the nearest‐neighbor MISR and MODIS points, since the spatial correlation length for cloud properties is of the order of tens or even a few hundreds of kilometers (Marchand, 2012). This choice is further justified later in this section. The mean geolocation difference for collocated pixels was found to be ∼0.4 km for both CATS‐MISR and CATS‐MODIS collocation. To find the collocated set of data, the following choices were made:

(1) Only those MISR data points are selected that lie within a distance of 380 km (MISR swath width) and whose observation time is within 5 min (to later accommodate MODIS granule time) from a given CATS point. From within this chosen subset of MISR data, a nearest‐neighbor search finds the nearest point lying within a 1‐km distance from the CATS data point, if any. If collocated points are found, only then is a MODIS search conducted.
(2) MODIS granules that lie within a 5‐min window of a given CATS‐MISR datum are selected for a nearest‐neighbor search. Given the fact that the MISR swath is swaddled within the MODIS swath, the MODIS nearest‐neighbor point also lies within 1 km of the CATS‐MISR datum, for all cases. When the point is found, MISR and MODIS CTH, MODIS CTH detection technique, and CATS cloud layer‐heights, associated 1,064 nm backscatter, surface elevation, and geolocation are extracted and stored. The altitude of the center of the highest lidar range‐gate having cloudy feature mask in a column is taken as the cloud‐layer height, whereas the base of the cloud‐layer is taken to be the height of the range‐gate, which is followed by at least 10 successive clear‐featured gates. Multiple layers can be sampled this way, but in our approach that follows, we shall be primarily focusing on the heights of the topmost one or two CATS cloud layer(s). In addition to the steps, we have taken to clearly distinguish cloud layers in our dataset, we shall still take cloud opacity estimates into account in examining cloud boundaries, as described in Section 4.

**Figure 1:**
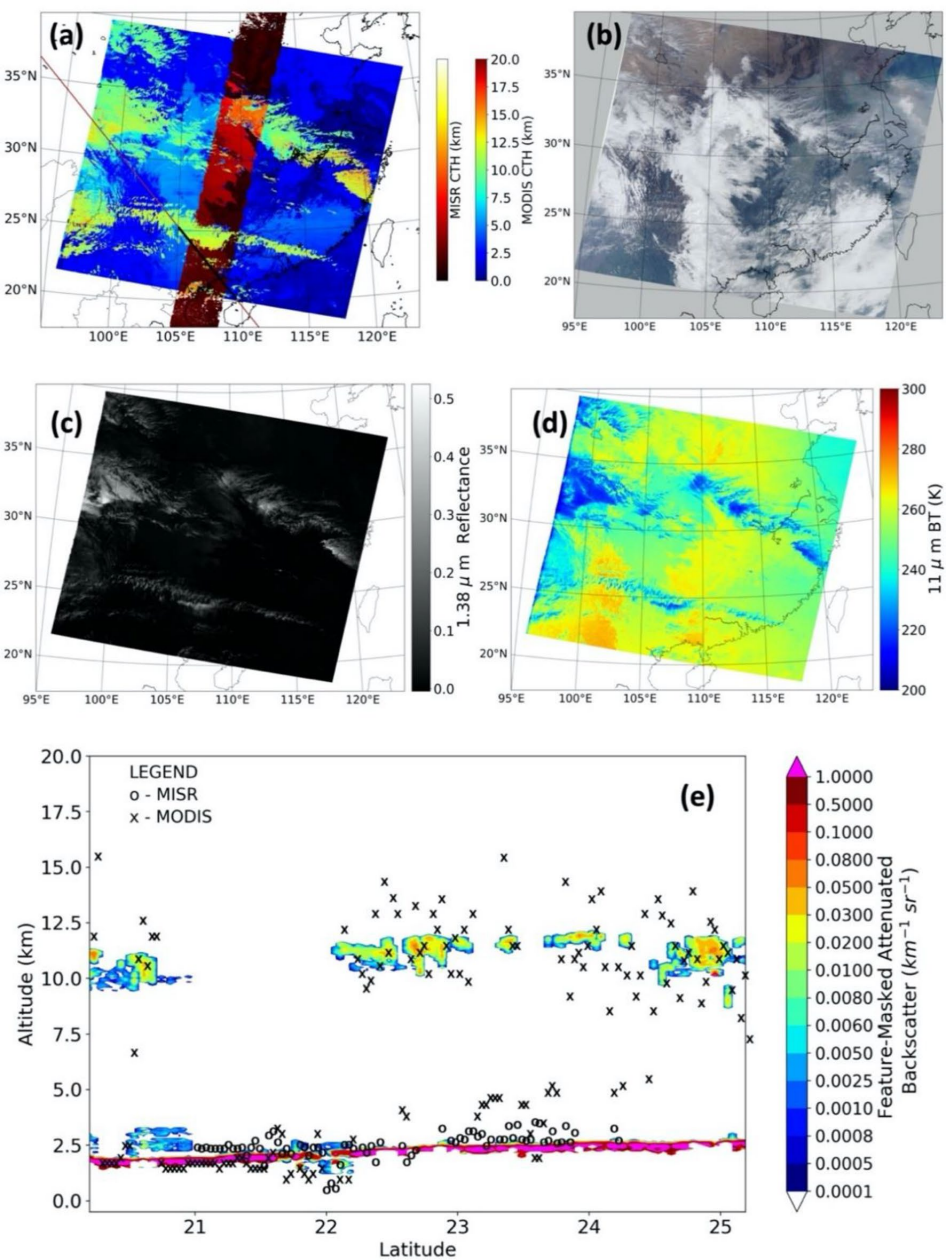
A collocation case from March 14, 2016 over South‐East Asia, between Moderate Resolution Imaging Spectroradiometer (MODIS), Multiangle Imaging Spectroradiometer (MISR), and Cloud‐Aerosol Transport System (CATS). (a) MODIS and MISR CTH. The part of the CATS orbit that was coincident with MISR and MODIS is in black. (b) MODIS RGB. (c) MODIS 1.38‐µm Reflectances (d) MODIS 11‐µm Brightness Temperatures (e) The vertical cross‐section of attenuated backscatter of cloud layers as detected from CATS. The collocated MISR and MODIS CTHs are also plotted. All CTHs are with respect to the WGS84 ellipsoid.

Figure 1 shows an example of successful collocation among all three instruments from March 14, 2016, over Southeast Asia. Figure 1a shows the three different swaths along with CTH from MISR and MODIS, with the collocated points marked in black. Figures 1b–1d shows the same scene in MODIS RGB, 1.38 µm reflectance, and 11 µm brightness temperature, respectively, whereas Figure 1e depicts the CATS‐retrieved vertical profile of cloud‐masked attenuated backscatter, along with collocated MISR and MODIS CTH. This particular scene was chosen as it has low‐lying cumuli, both with and without cirrus cover. For single‐layered clouds, as between 21°N and 22°N, there is greater agreement between MISR and MODIS CTHs. However, the presence of cirrus between 22°N and 24°N (see lidar in 1e, cooler cloud tops in 1 day and 1.38 µm imagery in 1c) leads to severe disagreements between MISR and MODIS, with MISR CTH consistently picking up the lower cumuli and MODIS retrievals being highly variable. The range wherein collocations are within the MISR swath extends from 21°N to 24°N. All heights are with respect to the World Geodetic System 1984 (WGS84) ellipsoid.

**Figure 2:**
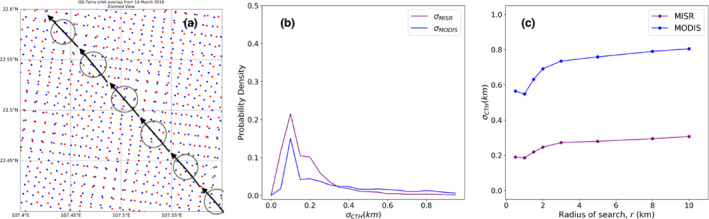
(a) A highly zoomed‐in view of the collocation case from 14 March 2016, between Moderate Resolution Imaging Spectroradiometer (MODIS), Multiangle Imaging Spectroradiometer (MISR), and Cloud‐Aerosol Transport System (CATS), from Figure 1. MODIS geolocations are in blue, MISR in red. The black arrows signify the general direction and 5‐km along‐track extent of the CATS pixels, with the dark circles approximately signifying 1‐km radii (not to scale) from the CATS geolocations, within which the nearest neighboring MISR and MODIS pixels were searched. (b) Normalized frequency of occurrence histograms of the standard deviation of MISR (purple) and MODIS (blue) CTHs from within each 1‐km radius search windows for all successful collocations for the year 2016. (c) Mean standard deviation of MISR (purple) and MODIS (blue) CTH for progressively bigger search radii, for all successful collocations from 2016.

An intuitive sense for the collocation process can be formed from Figure 2. Figure 2a shows a highly zoomed‐in view of a patch of MISR and MODIS geolocations from the same scene as in Figure 1, with a set of CATS pixels cutting through. The search for collocated data is conducted within the 1‐km radii circular windows that are marked around each CATS geolocation in Figure 2a (the circles are merely representative and not to scale). With navigation errors (∼100 m), collocation differences (∼400 m), and mismatches in pixel‐size among instruments (∼1 × 1 km vs. ∼14 m × 5 km), it is the local CTH variations below these scales that introduce uncertainty in comparing MISR or MODIS CTH with CATS. To quantify this random error, we found all the MISR and MODIS data that lay within circular regions for each of the 9,538 CATS points that satisfied the co‐location conditions for the year 2016 and examined CTH variations as a function of radius of the circular region. For example, the histograms of the standard deviations in CTH within each region of 1‐km radius (number of neighbors at least 2), denoted as σ_MISR_ for MISR and σ_MODIS_ for MODIS, are shown in Figure 2b. Both σ_MISR_ and σ_MODIS_ peak at 0.1 km, with their mean values being 0.2 and 0.5 km, respectively. Thus, the CTH of each collocated point from MISR and MODIS can be taken to be generally representative of CTH of all other observations within a 1‐km radius circle centered around the CATS data point, with an uncertainty of about 200 m for MISR and 500 m for MODIS. There is also a mismatch in resolution between MISR/MODIS (∼1 km) and CATS (5 km), as well as wind displacement of clouds during the maximum allowed time‐interval between observations of 5 min in our coincidence criteria (e.g., a high wind speed of ∼30 m/s can displace clouds close to 10 km in 5 minutes). Thus, local CTH variations over scales up to ∼10 km also introduce uncertainty in comparing the CTH between MISR or MODIS with CATS. Thus, σ_MISR_ and σ_MODIS_ are calculated for progressively increasing search radii up to 10 km and plotted in Figure 2c. It is observed that both σ_MISR_ and σ_MODIS_ exhibit asymptotic behavior with increasing distances, reaching 0.3 and 0.8 km, respectively. These values can be interpreted as an upper limit of CTH error owing to our method of collocation. The error is larger for MODIS because MISR is generally more sensitive to lower clouds (owing to the higher spatial contrast they offer relative to thin cirrus) than MODIS, where variability in CTH and emissivity are smaller compared to high and midlevel clouds‐evident, for example, in Figure 1e. When segregated by CTH (not shown), collocation errors are generally found to be higher for high clouds (CATS CTH > 5 km), with σ_MISR_ and σ_MODIS_ for a 10‐km search‐radius being 0.5 and 0.9 km, respectively. On the other hand, σ_MISR_ and σ_MODIS_ for a 10‐km search‐radius for low clouds (CATS CTH < 5 km) are 0.3 and 0.5 km, respectively. These findings suggest that collocation errors are a more pertinent issue in analyzing errors in high cloud heights as compared to low clouds, from both MODIS and MISR. One might consider the possibility of filtering data based on some pre‐set threshold on MISR or MODIS observed CTH variability, but such an exercise would be tantamount to presupposing MISR and MODIS CTH uncertainty; hence, it is avoided.

In most of this study going forward, the topmost CATS cloud layer height is compared against MODIS and MISR CTH, since these operational passive sensors only retrieve a single CTH per pixel. However, we also recognize that passive sensors may be sensitive to the tops of lower cloud layers that are underlying thin upper clouds. Therefore, to investigate the sensitivity of sensors to individual layers, the closest CATS layer to MISR/MODIS CTH is also studied in Section 4.4. This study aims to understand and quantify the uncertainty in MODIS and MISR CTHs, be it the height of the topmost cloud layer or the height of lower cloud layers underlying thin upper clouds and the conditions in which passive sensors retrieve CTH that are consistent with lidar‐retrieved top cloud layer.

## 4 Results and Causes of CTH Differences

By applying the collocation method described above, 33 months (February 2015–October 2017) of collocated MISR, MODIS, and CATS CTHs have been compared spanning a semi‐global domain (between 50°N and 50°S). In total, 51,622 collocated (clear + cloudy) points were collected, among which, 27% were rejected as flagged clear by MODIS; 12% were outside the region of MISR swath with valid retrievals; 22% reported MISR CTH “no‐retrievals,” that is, MISR stereo failed owing to a lack of contrast (e.g., over clear sky ocean); and 2% did not have valid CATS cloud‐layer retrieval where MODIS and MISR retrieved a CTH. Over land (provided enough surface texture), MISR stereo can retrieve surface elevation as stereo height. Such features have been dealt with in our study by subtracting surface elevation from MISR stereo heights for every collocated point and further only retaining such points in our analysis whose surface‐elevation‐corrected stereo heights were at least greater than 562 m–the value used by MISR for cloud designation (Mueller et al., 2013). The CATS pixel‐level surface elevation from the 1 × 1 km USGS GMTED2010 digital elevation map (DEM) is used for this purpose. Finally, our analysis on valid CTH retrievals was conducted on a dataset of 18,986 cloudy points.

### 4.1 Global and Regional Biases From MISR, MODIS, and CATS Inter‐Comparison

**Figure 3:**
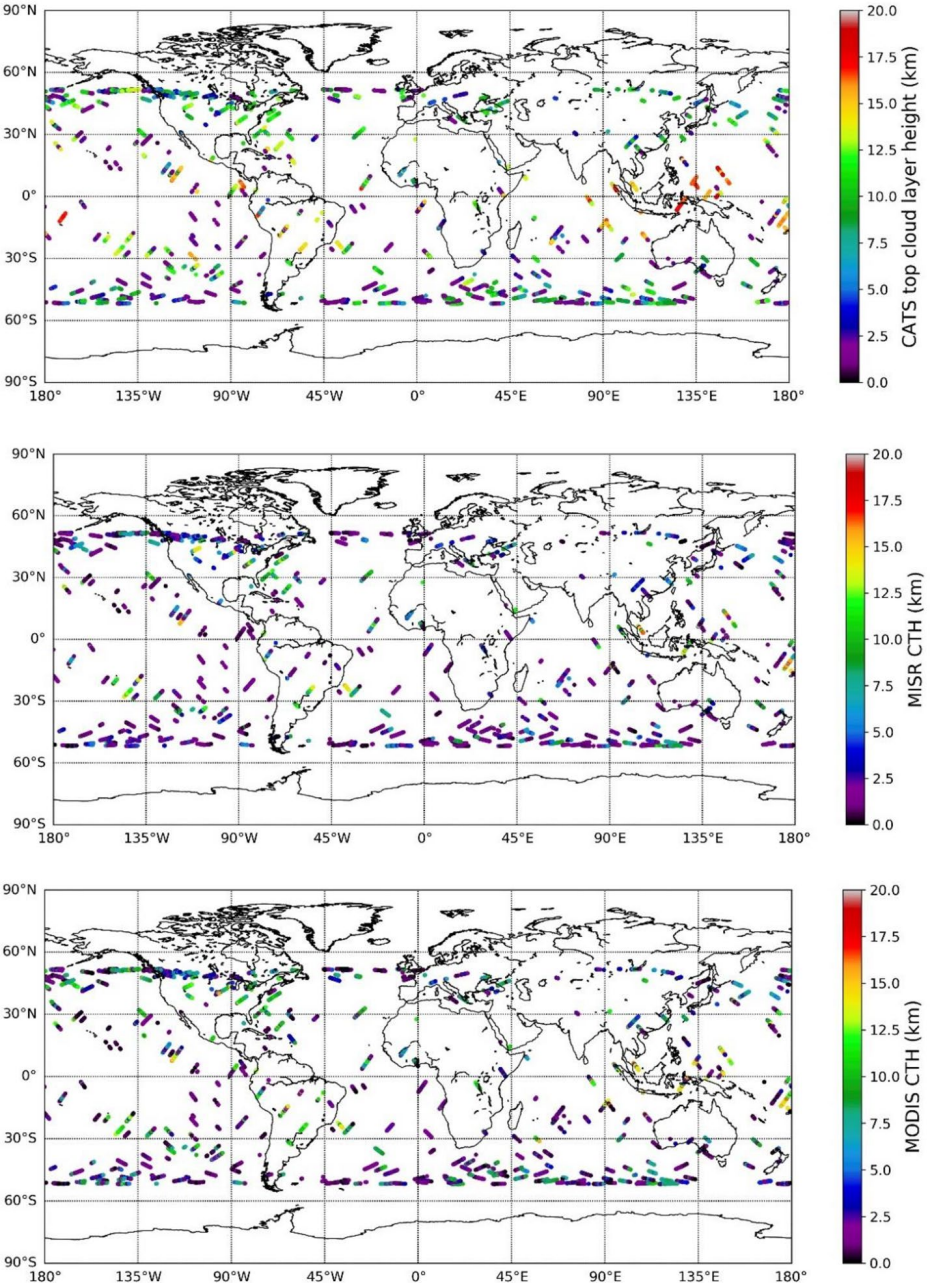
The global distribution of collocations of Cloud‐Aerosol Transport System (CATS), Multiangle Imaging Spectroradiometer (MISR), and Moderate Resolution Imaging Spectroradiometer (MODIS), where all three instruments recorded valid cloud top height retrievals for all of CATS operation. Eighteen thousand nine hundred and eighty‐six individual collocated points have been plotted in the figure.

Figure 3 shows the global distribution of all 18,986 collocated CATS, MISR, and MODIS CTHs. Unless otherwise noted, CATS CTH will refer to the topmost CATS cloud‐layer altitude. Figure 3 shows that there is a much higher frequency of collocation near the 50° latitudes in both hemispheres, due to greater swath overlap of Terra with ISS. This study is restricted to an inter‐comparison over the tropics and midlatitudes since the ISS orbit does not venture further poleward. Also, Figure 3 shows that CATS detects the presence of a lot more very high CTH (e.g., West Pacific warm pool region) than MODIS or MISR, owing to the lidar’s ability to detect optically thinner clouds. From detailed examination of Figure 3, one notices that MISR detects more low CTHs than CATS or MODIS, because MISR stereo is sensitive to spatial texture in multi‐angular views, which is greater for lower, textured clouds, even under cirrus. The textured nature of the radiance field in the Western Pacific warm pool was recently examined by Hong and Di Girolamo (2020), demonstrating that the texture of ice‐above‐liquid clouds was only slightly smoother than liquid‐only clouds owing to the fact that cirrus in the region are generally optically thin. Hence, the spatial contrast observed by MISR has the largest contribution from liquid clouds under conditions of ice‐over‐liquid clouds in the region.

**Figure 4:**
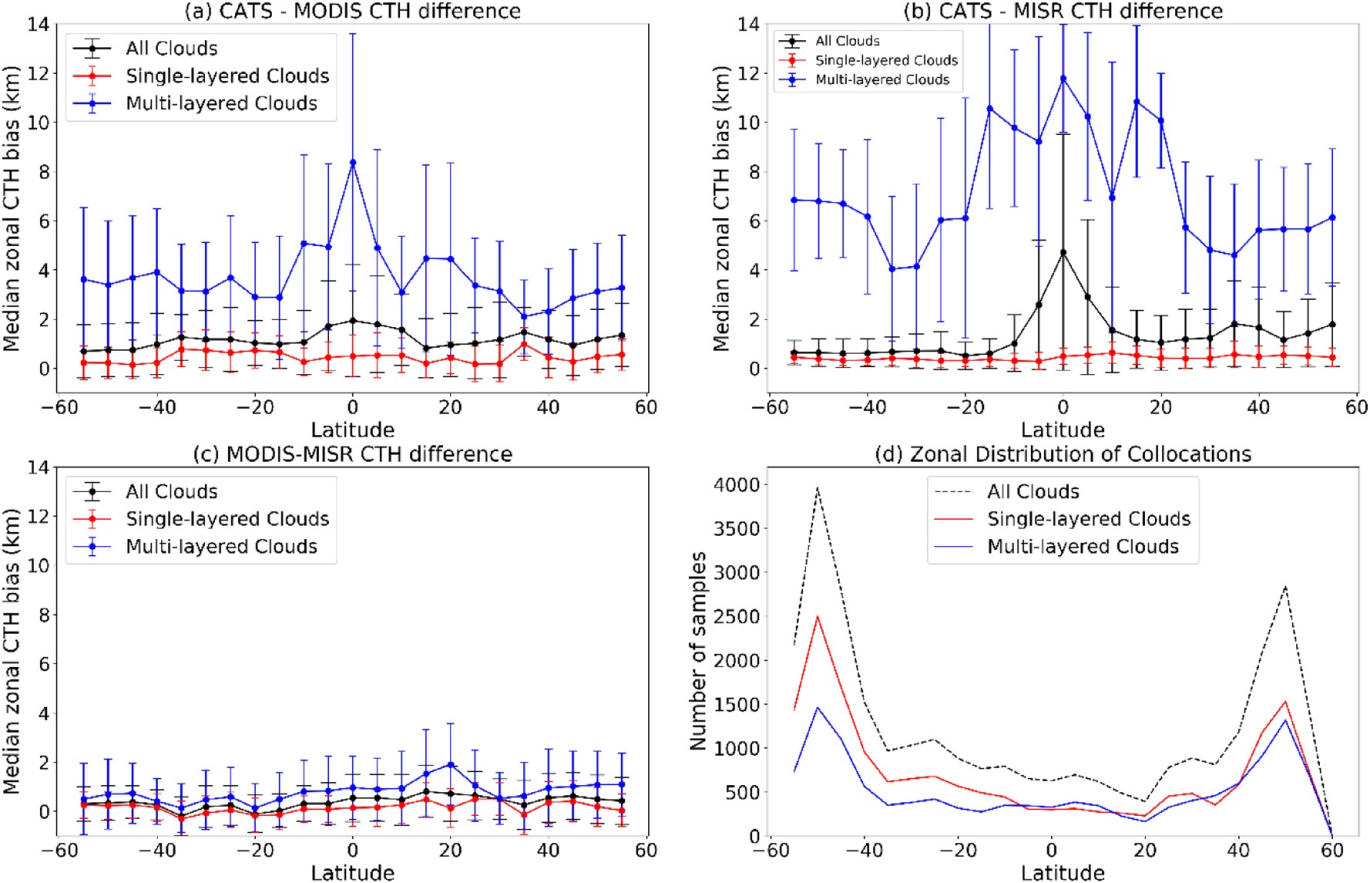
Zonal distribution of median CTH differences for Cloud‐Aerosol Transport System (CATS)‐Moderate Resolution Imaging Spectroradiometer (MODIS) (top left), CATS‐Multiangle Imaging Spectroradiometer (MISR) (top right), and MODIS‐MISR (bottom left) for all clouds (black), CATS single‐layered clouds (red) and CATS multi‐layered clouds (blue). The number of samples in each zonal bin (5° interval) is shown in the bottom right panel. Error bars in the first three subplots represent the median absolute deviation statistic.

Figure 4 shows the zonal dependence of CTH differences between the three instruments, expressed as (a) CATS‐MODIS, (b) CATS‐MISR, and (c) MODIS‐MISR. In each individual panel, the median CTH difference for every 5° latitude interval from 60°N to 60°S was plotted at the mid‐point of each corresponding interval. Each figure shows the median CTH difference for the bin for all clouds in black, CATS single‐layered clouds in red, and multi‐layered clouds in blue. The error bars for each point signify the median absolute deviation, a robust statistic that is directly proportional to statistical dispersion but is resilient to the presence of outliers in a non‐normal distribution. For CATS‐detected multi‐layered clouds, there are at least two cloud layers present, with the layers being separated by a vertical distance of at least 600 m (10 range‐gates). The last panel (d) depicts the zonal distribution of number of samples. As can be seen from Figures 4a–4c, the largest differences in median CTH for all clouds (in black) are observed about the equator in the tropical regions (between 20°N and 20°S), owing to the contribution from multi‐layered clouds. Large differences near the tropics were also noticed in the CALIOP and Aqua MODIS CTH difference record by Holz et al. (2008) and is due to the frequent presence of high and optically thin cirrus, often overhanging low and optically thick cumuli (e.g., Li et al., 2015; Stubenrauch et al., 2013). Moreover, from Figure 4, the median deviations for both CATS‐MODIS and CATS‐MISR CTH for multi‐layered scenes are much greater than single‐layered clouds. This increase for multi‐layered scenes is more pronounced for CATS‐MISR than for CATS‐MODIS, because MODIS and CATS are theoretically more sensitive to higher clouds under cloud overlap, whereas, MISR is more sensitive to textured low clouds, even in the presence of overlying optically thin cirrus (e.g., Naud et al., 2007). The increase in median CTH for MODIS for multi‐layered clouds is smaller than for MISR, and this can be attributed to MODIS underestimating the semi‐transparent top layer height, when the lower layer is optically thick (Menzel et al., 2015). The increase of median CTH differences for multi‐layered clouds is consistently <2 km for MODIS‐MISR, suggesting median MODIS and MISR CTH are closely similar; this will be explored in upcoming sections.

We note that Figure 4d should not be interpreted as a proper zonal climatology of cloud overlap. This is because our data are strictly limited to cases where we have CATS, MODIS, and MISR detecting and reporting CTHs, as well as other sampling issues not discussed here. Still, dividing the number of samples of multi‐layered clouds to all clouds in Figure 4d leads to a result that is reasonably similar to CALIPSO results from Figure 2b of Yuan and Oreopoulos (2013), recognizing the different diurnal sampling times of Terra and CALIPSO.

### 4.2 Height of the Top Cloud Layer

**Figure 5:**
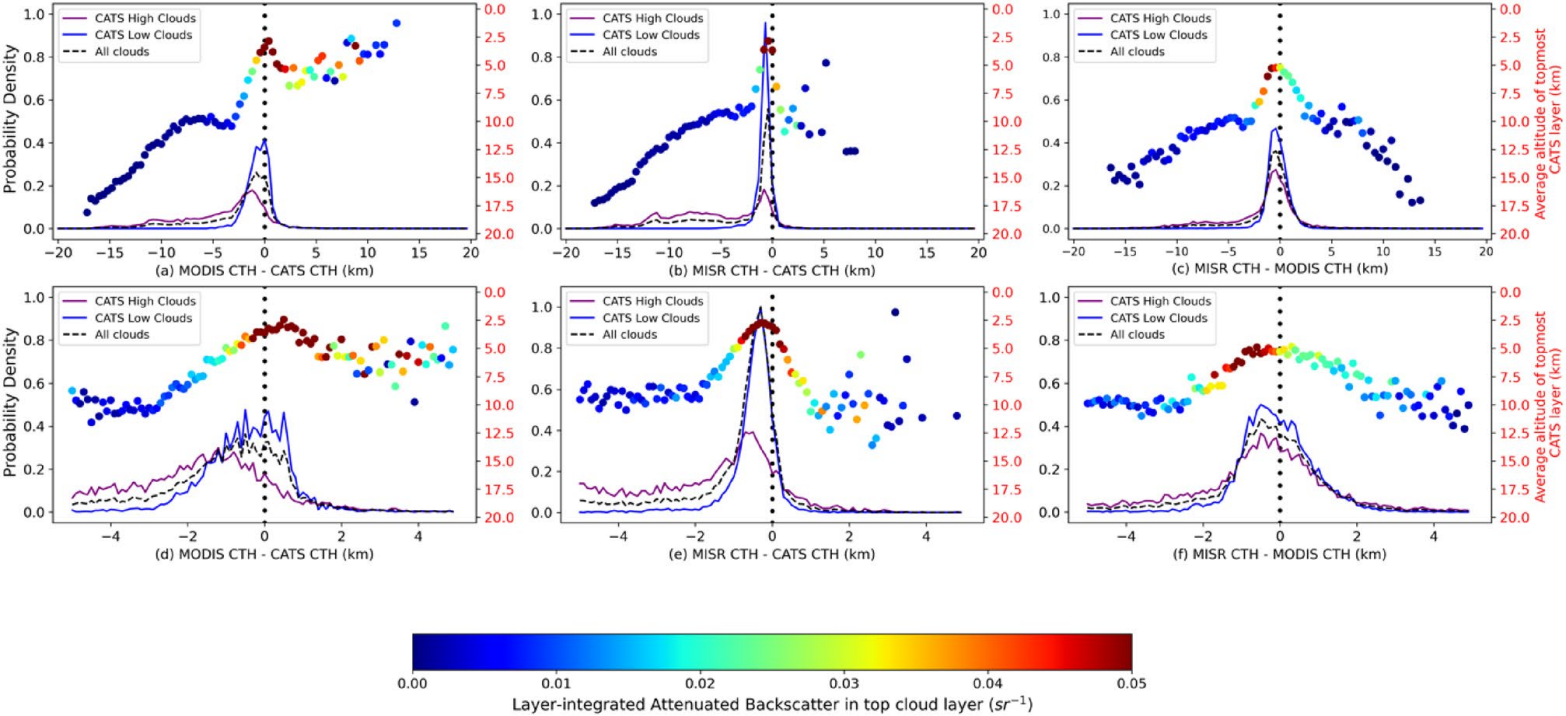
Normalized frequency of occurrence histograms of cloud‐top heights (CTH) differences for high clouds [Cloud‐Aerosol Transport System (CATS) CTH > 5 km, purple lines] and low clouds (CATS CTH < 5 km, blue lines), with 100 bins between +20 km and −20 km (top panels) and +5 km and −5 km (bottom panels). The normalized frequency of occurrence histograms for the overall distributions are marked by black dashed lines and contain 18,986 collocated data points, out of which 10,315 are high clouds. The mean of the CATS cloud‐top heights for all top layers in a histogram bin is represented by a large colored dot and is associated with the red *y*‐axis to the right of each panel. The color of the dot itself represents the CATS layer‐integrated backscatter (γ) for the topmost cloud layer.

To further investigate CTH differences, histograms for the three instrument pairs have been plotted in Figure 5. The CTH differences here are (a and d) MODIS‐CATS, (b and e) MISR‐CATS, and (c and f) MISR‐MODIS, respectively. 100 equal‐sized bins between −20 and 20 km, and between −5 and 5 km, have been used for the top‐ and bottom‐panel, respectively, with all histograms centered at zero. CATS CTH is the topmost CATS layer height. While analyzing these results, one must be mindful that different instruments’ CTH might be due to cloud occurrence at different altitudes; this issue of cloud overlap in the interpretation of CTH differences is examined in Section 4.4. In Figure 5 and in figures to follow, an inverted system of axes in red has been added showing mean CATS top‐layer height in each histogram bin, each point further color‐coded by mean CATS top‐layer layer‐integrated backscatter (*γ*), for all scenes in that bin. A lower *γ* denotes an optically thinner cloud. A CATS *γ* = 0.02 sr^−1^ approximately corresponds to mean layer‐integrated optical depth (OD) of 0.8 (linear regression between CATS Level 2 OD with integrated backscatter). In each Figure 5 subplot, the purple line signifies CATS high clouds (CTH > 5 km), the blue line signifies CATS low clouds (CTH < 5 km), while the dashed black line signifies all collocated points. Of these 18,986 points, 10,315 were high clouds and the rest low clouds.

Figure 5 shows that high negative CTH differences in the MODIS‐CATS and MISR‐CATS distributions, and high absolute CTH differences in MISR‐MODIS distribution arise from the presence of optically thin, high cloud layers. Very high positive (>2 km) MODIS‐CATS and MISR‐CATS CTH differences occur much less frequently than high negative differences. The frequency of these very high positive cases is rare, occurring in two independent collocation incidents for MISR and for 5 collocation incidents for MODIS (thus, much less than 1% of all cases for both). Such low occurrences are consistent with expectations of collocation errors computed from the results of Figure 2c, even under the worst‐case and unrealistic assumption that all the variability is due to actual height variability rather than CTH retrieval noise. The peaks of the distributions for all clouds (dashed black) in the top panel are at (a) −800 m for MODIS‐CATS, (b) −420 m for MISR‐CATS, and (c) −80 m for MISR‐MODIS. These peaks exist where γ is the largest. There exist prominent tails in all distributions on the left side of the distribution modes, extending up to about −15 km for MODIS‐CATS and MISR‐CATS and up to −12 km for MISR‐MODIS. These long tails are due to optically thin, high clouds, with *γ* < 0.02 sr^−1^ and with mean CATS top‐layer height of > 10 km. Most cases in the MODIS‐CATS (76%) and the MISR‐CATS (89%) distributions involve negative CTH differences (i.e., MODIS and MISR CTH below CATS top‐layer height). Most positive MODIS‐CATS differences are for scenes with CATS top‐layer height below 7 km and *γ* > 0.03 sr^−1^ (OD ∼ 1.2). Most positive MISR‐CATS differences, however, arise from mid‐level clouds (CTH ∼ 5–8 km), and moderate optical thickness (*γ* ∼ 0.02 sr^−1^, OD ∼ 0.8). These positive offsets are discussed in more detail later in the manuscript (Sections 4.3 and 4.5.2).

As evident in Figure 5 and in other figures to follow, CTH differences exhibit a well‐defined mode that are offset from zero, with a fat tail to the left of the mode. The mode arises from the inherent uncertainties in the retrieved CTH and collocation of instruments, whereas the fat tail arises from the differing sensitivity of the instrument techniques at detecting the height of different cloud layers under multi‐layered conditions (which will become apparent in Sections 4.3 and 4.4). Separating these two effects is necessary for a proper understanding and quantitative description of the error budget. To do so, we note in Figure 5, and in others figures to follow, that the mode appears approximately Gaussian from its maximum down to its half‐maximum value. It also appears approximately Gaussian over the entire right‐side of the mode. Therefore, rather than providing a Gaussian fit to the entire data distribution, which would be seriously impacted by the fat left‐side tail, we use the Gaussian solution for the mode standard deviation, given by σ =  $\frac{\mathrm{FWHM}}{2\sqrt{2\;\ln\;2}}$, where the FWHM is the Full Width at Half‐Maximum for our distribution. In this way, the mode off‐set from zero (i.e., the mode bias) and mode standard deviation can be used to describe the uncertainties inherent in the retrieved CTHs and collocation of instruments, with the remaining difference from this Gaussian on the fat left‐side tail attributed to the differing sensitivity of the techniques at detecting the height of different cloud layers under multi‐layered conditions. We have also examined estimates of mode standard deviation by replacing the FWHM with the Half‐Width at Half‐Maximum (HWHM) for both sides of the mode (and multiplying by 2) and found the estimates of mode standard deviation from the two approaches diverge by less than 50 m for all the distributions that will follow in this paper. Therefore, we report only the mode standard deviation from the FWHM approach.

Apart from the mode bias and mode standard deviation, an upper bound on the sampling uncertainty is nominally estimated for all instances in this paper by simply computing standard errors using the number of orbit‐segments as independent estimates. In all instances to follow, the standard error is always less than 10 m; hence, the sampling uncertainty is low (≤5% of 1σ) in our study and not reported separately for all instances.

For high cloud scenes (purple lines, Figure 5), there is much disagreement between the three instruments. From Figures 5a and 5d, MODIS high‐cloud CTH mode bias = −1,160 m and precision = 1,080 m, while from Figures 5b and 5e, MISR high cloud mode bias = −540 m and precision = 590 m. This difference in the MODIS and MISR errors arises primarily from scenes where multiple cloud layers are present, and the instruments are identifying different layers to report height (explored further in Section 4.4), with MISR being more likely to retrieve the height of the lower cloud when thin cirrus is also present, while MODIS CTH is dependent on optical and geometrical properties of the multiple cloud layers in the scene (Holz et al., 2008; Naud et al., 2002, 2007; Stubenrauch et al., 2013). Further MODIS errors arise due to optically thin but geometrically thick cirrus, as the assumption of a geometrically thin cloud layer is central to the effectiveness of CO_2_‐slicing (see Sections 4.3 and 4.5.2). The probabilities of MISR and MODIS detecting the true height of a CATS high cloud to within ±1 km are nearly equal at about 15%, in spite of MISR not being as sensitive as MODIS to optically thin cirrus. MODIS underestimation of high CTH for multi‐layered scenes seems to be the primary reason behind this phenomenon (see Section 4.4).

There is much agreement between the instruments for low clouds (blue line, Figure 5). From Figures 5b and 5e, MISR‐CATS CTH difference exhibits a sharp distribution, with MISR low‐cloud mode bias = −320 m and precision = 250 m. MISR‐CATS low cloud CTH differences fall within 0 and −2 km 88% of the time, with positive CTH differences constituting 9% of all cases and the occurrence of CTH differences <−2 km being even more rare at 3%. In comparison, MODIS low cloud CTH (Figures 5a and 5d) exhibits a mode bias = 40 m and precision = 730 m, with 14% of MODIS‐CATS differences below −2 km and 29% of differences above 0. For low clouds, MODIS uses the IR BT technique with latitudinally varying climatological lapse rates (Baum et al., 2012). Significant deviations from these lapse rates are source of uncertainty. Holz et al. (2008) and Harshvardhan et al. (2009) demonstrated that the Collection five MOD06 product was overestimating CTH by over 2 km in cases where a low‐lying liquid phase cloud was present over the ocean, particularly in the presence of strong temperature inversions, due to poor representation in ancillary data. As rectification, the Collection six MOD06 algorithm started using zonally averaged “apparent 11‐μm brightness temperature (BT) lapse rates” from a combination of CALIOP CTHs and modeled sea‐surface temperatures to better capture boundary‐layer lapse rates (Baum et al., 2012). This improvement manifests itself in the absence of the hump in positive MODIS‐CATS differences that was observed in MODIS‐CALIPSO differences reported in Figure 8 of Holz et al. (2008).

Despite MISR applying stereoscopy and MODIS a radiometric technique, the two passive sensors do produce reasonable agreement in CTH. The MISR‐MODIS CTH difference distribution (Figures 5c and 5f) has mode = −400 m and mode *σ* = 680 m. 62% of all MISR‐MODIS CTH differences lie between ±2 km, and for this subset of scenes, the topmost cloud layer from CATS is optically thick (OD ∼ 1.5) at a mean altitude ∼5 km. The spread of the distribution is attributable to the natural variability of clouds in a scene and the different sensitivities of MISR and MODIS to this variability (Section 3). About 36% of CTH differences is constituted by differences between 0 and −2 km, mostly for top layers of cloud with integrated backscatter larger than 0.02 sr^−1^ and with heights <10 km and is associated with MODIS IR BT CTH overestimation for stronger temperature inversions (note, IR BT technique is applied for all but high and mid‐level ice clouds). A sizable portion of MISR–MODIS differences in both high and low cloud scenes (25% and 36%, respectively) has positive values up to +2 km. Positive MISR‐MODIS bias (mean difference = 0.6 km) for optically thin clouds is primarily due to optically thin and geometrically thick cirrus (mean geometric depth of top layer in the 0 to +2 km interval from CATS ∼ 1.2 km), and this role of OD on bias will be explored in the next section.

### 4.3 Optical Depth of the Top Cloud Layer

Figure 5 suggests that as one moves from large negative top‐layer CTH differences to zero, there is a general tendency of the top‐cloud layer to be lower and optically thicker for MISR and MODIS. As one moves from zero to positive CTH differences, the top layer starts to be slightly higher, with only a modest reduction in the mean backscatter. These tendencies are consistent with our knowledge of the three CTH retrieval techniques. This is especially true for CATS and MODIS, because their retrievals are highly dependent on cloud optical properties. An optically thicker top layer of cloud represents an opaque or a nearly opaque atmospheric column to the lidar, which leads to rapid attenuation of the lidar signal near the cloud top. This represents a strongly emissive cloud‐top layer; hence, the retrieved CTP (in case of the CO_2_‐slicing technique) or the CTT (in case of the 11µm‐BT technique) is very close to actual values. However, for more transmissive cases, CTT and BT can diverge substantially, resulting in lower CTH under typical conditions and higher under atypical conditions (i.e., surface or lower cloud layer being cooler than the cloud‐top layer). The CO_2_‐slicing approach hinges on an assumption of a thin cloud layer, and any geometrical depth (especially accompanied by low optical depth) can lead to underestimation of CTH, through an overestimation of CTP. Smith and Platt (1978) estimated errors of ∼50 hPa in CTP for a cloud of ∼100 hPa depth, and CO_2_‐slicing is generally likened to a center of mass problem (Menzel et al., 2008), with CTP errors co‐varying with optical depth into the cloud (i.e., CTP close to true cloud‐top for optically thick cases and closer to the geometric center for optically thin cases). As a result, the CTH difference in these cases is a function of the vertical distribution of extinction in the cloud layer, as well as temperature throughout the column. On the other hand, although MISR makes use of a stereoscopy, MISR‐CATS differences are also expected to depend on the vertical distribution of single scattering properties of the top cloud layer, as well as its horizontal distribution that gives rise to the spatial contrast for stereoscopy to work. For a single layer cloud, the contrast is expected to emerge over some depth of the cloud layer that is likely deeper than a lidar‐derived height. For an optically thin upper cloud overlapping an optically thick lower cloud, the largest spatial contrast may well emerge from the lower cloud layer, allowing stereo to retrieve the CTH of the lower cloud layer. The exact relationship of this “*stereo‐opacity bias*” with the 3D distribution of cloud optical properties has yet to be quantified from theory or experiments.

**Figure 6:**
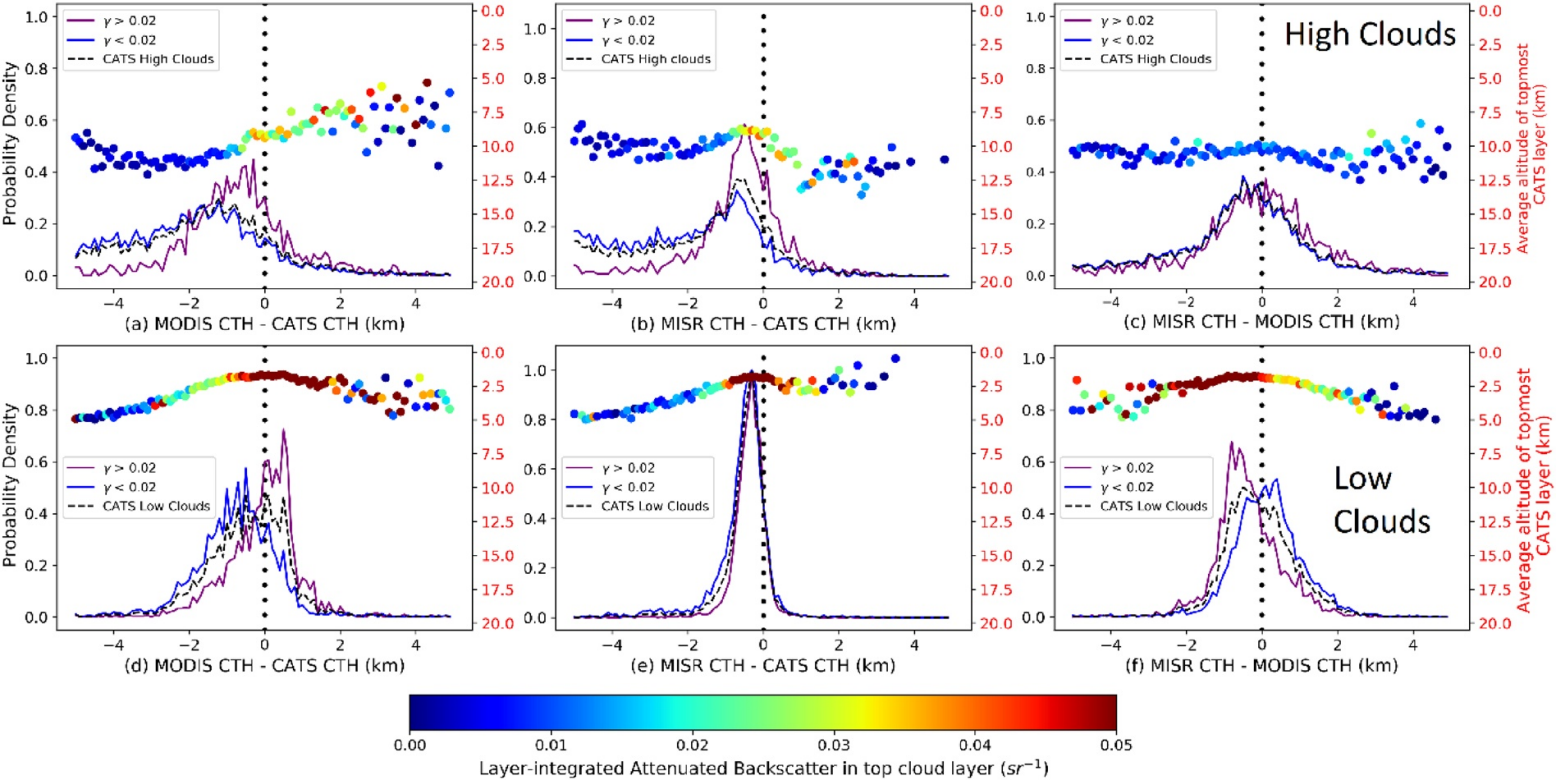
Normalized frequency of occurrence histograms of cloud‐top heights (CTH) differences for (a–c) high clouds and (d–f) low clouds from Cloud‐Aerosol Transport System (CATS), for optically thick top layer of cloud (*γ* > 0.02 sr^−1^, purple lines) and optically thin top layer of cloud (*γ* < 0.02 sr^−1^, blue lines), with the black dashed lines denoting overall distributions. The mean of the CATS cloud‐top heights for all top layers in a histogram bin is represented by a large colored dot and is associated with the red *y*‐axis to the right of each panel. The color of the dot itself represents the CATS layer‐integrated backscatter (*γ*) for the topmost cloud layer.

To gauge the impact of the top‐layer cloud optical properties on the retrieval of CTH for low and high clouds from MODIS and MISR, Figure 6 shows histograms of CTH differences for the three instrument pairs with 100 equal‐width bins between −5 km and +5 km, for optically thick top cloud layers (*γ* > 0.02 sr^−1^) in purple and optically thin top cloud layers (*γ* < 0.02 sr^−1^) in blue. The top panel (Figures 6a–6c) is for CATS high clouds (CATS CTH > 5 km), while the lower panel (Figures 6d–6f) is for CATS low clouds (CATS CTH < 5 km). Based on the observed relationships between CTH differences and backscatter in the previous figures, *γ* = 0.02 sr^−1^ (OD∼0.8) is simply chosen as a distinction between optically thick and optically thin cloud.

From Figure 6a, the MODIS‐CATS CTH difference for optically thin, high topmost cloud layer shows much variance around the mode, especially for negative differences. The issues faced by the CO_2_‐slicing technique for semi‐transparent clouds are many‐fold, including errors due to cloud geometrical depth and the presence of lower cloud layers (Smith & Platt, 1978; Wielicki & Coakley, 1981; Wylie & Menzel, 1989), leading to MODIS optically thin high clouds mode bias = −1,200 m and precision = 1,020 m, with 84% of MODIS‐CATS CTH differences being negative. MODIS optically thick high cloud CTH has mode bias = −280 m and precision = 730 m, and the MODIS‐CATS distribution has a sharper peak. From Figure 6b, MISR optically thick high cloud mode bias = −440 m and precision = 470 m, with the MISR‐CATS distribution being less noisy than the corresponding MODIS‐CATS distribution. The distributions for optically thick (mode = −20 m, mode *σ* = 610 m) and thin high clouds (mode = −320 m, mode *σ* = 640 m) for the MISR‐MODIS difference (Figure 6c) are both symmetrical about their modes.

For CATS low clouds, from Figure 6d, the MODIS‐CATS distribution shows two distinct peaks–the optically thin cloud distribution (mode bias = −440 m and precision = 600 m) and the optically thick cloud distribution (mode bias = 500 m and precision = 430 m) are both consistent with the limitations of the IR BT technique and with the Collection six improvements. Optically thin clouds being more transmissive allow more IR radiation from closer to the warm surface to reach the satellite, leading to negative CTH bias, whereas the positive bias for optically thicker or more emissive clouds presumably owes its origin to a larger deviation of true boundary‐layer lapse rates from the Collection six climatological lapse rates. However, it needs to be noted that the bias for both optically thin and thick low clouds shows a marked improvement from Collection five (Figure 11b of Baum et al., 2012). The MISR‐CATS distributions (Figure 6e) for optically thick low clouds (mode bias = −280 m and precision = 260 m) and optically thin low clouds (mode bias = −320 m and precision = 310 m) exhibit a slight dependence of MISR low cloud retrieval on the optical depth (see discussion above). This will be explored in the next section after we have quantified the relationship of CTH differences with multi‐layering. The distributions for optically thick and thin low clouds for the MISR‐MODIS difference (Figure 6f) closely resemble that of the MODIS‐CATS CTH distributions as the dependence of MISR CTH on OD is considerably lesser than that of MODIS CTH.

To recap, the previous two sections have quantified CTH differences between sensors, examined how these differences depend on the top layer properties, and provided evidence of significant contribution from cloud overlap in explaining these differences. The next section isolates those contributions and, in their absence, examines the depth within the cloud that these instruments are most sensitive to.

### 4.4 Multi‐Layered Clouds

**Figure 7:**
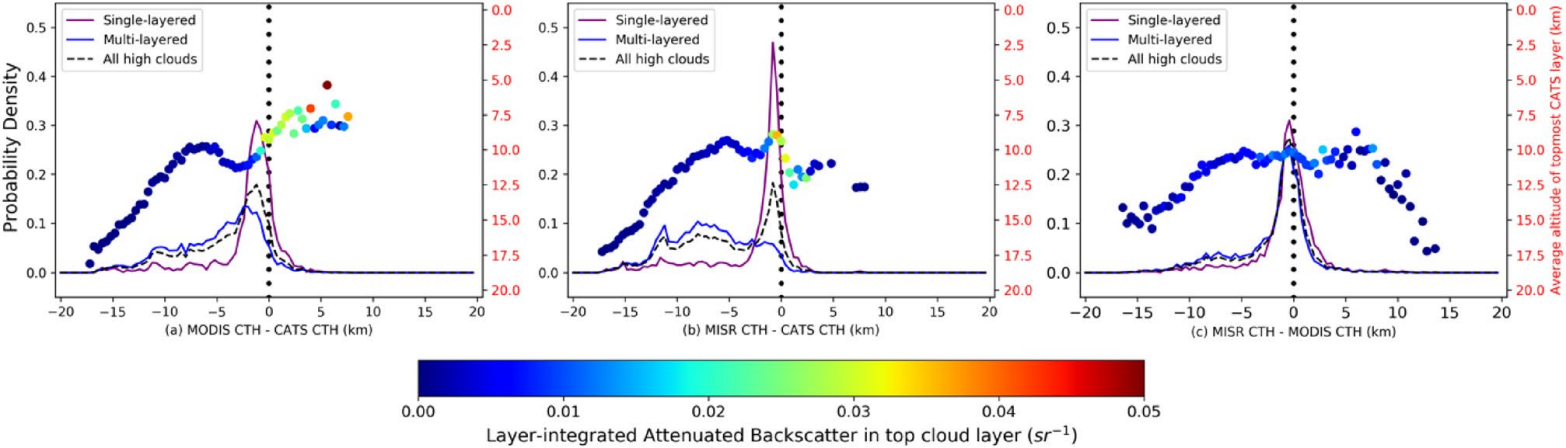
Normalized frequency of occurrence histograms of cloud‐top heights (CTH) differences for Cloud‐Aerosol Transport System (CATS) single‐layered clouds (CATS Percentage Opacity <50%, purple lines) and multi‐layered clouds (CATS detected at least two layers, blue lines), with the CATS top layer height being greater than 5 km. The mean of the CATS cloud‐top heights for all top layers in a histogram bin is represented by a large colored dot and is associated with the red *y*‐axis to the right of each panel. The color of the dot itself represents the CATS layer‐integrated backscatter (γ) for the topmost cloud layer.

Past research (Holz et al., 2008; Marchand et al., 2007; Naud et al., 2004, 2007) and the previous sections have flagged multi‐layered clouds as leading to passive sensor CTH errors. To quantify this, histograms of CTH differences are shown in Figure 7, based on multi‐layering for CATS high cloud (CTH > 5 km), with 100 equal‐sized bins between −20 and 20 km. The purple line indicates single‐layered high cloud, the blue line indicates at least more than one layer (with minimum vertical separation of 600 m), and the black line is a histogram for all high clouds. Moreover, since an optically thick high cloud can completely attenuate the lidar signal (preventing low‐cloud detection), we further restrict single‐layered clouds to those scenes with CATS Percentage Opacity lesser than or equal to 0.5. CATS reports a Percentage Opacity, defined as the fraction of “opaque” (no surface detection) 350 m‐resolution Level 1 samples that constitute a Level 2 5‐km datum. A value of 0 signifies “all profiles transparent,” while 1 signifies “all profiles opaque.” Note, the term “Percentage Opacity” should not be confused with a measure of cloud optical depth; rather it should be thought of as a measure of the sub‐pixel transmittance homogeneity of a CATS datum. This threshold is applied to reduce the occurrence of multi‐layered broken clouds, which can make comparisons between the different product resolutions tenuous. The mode bias reported in the rest of Section 4.4 is largely insensitive to this threshold; but MISR and MODIS CTH mode precision for all clouds deteriorate by 60 and 80 m, respectively, when all cloud samples are accepted without a threshold on Percentage Opacity.

From Figure 7, the greatest occurrence of negative MODIS‐CATS (Figure 7a) and MISR‐CATS CTH differences (Figure 7b) is found in multi‐layered cases where the top‐layer has *γ* < 0.02 sr^−1^ and a mean CTH of more than 10 km. In multi‐layered cases, CATS top‐layer CTH and MODIS CTH are within 1 km of each other 10% of the time, while it is only 4% for CATS top‐layer CTH and MISR CTH. Compared to that, negative differences less than −2 km are observed in 7% of all single‐layered cases in the MODIS‐CATS distribution (mode bias = −1,160 m and precision = 510 m) and a total of 5% in the MISR‐CATS distribution (mode bias = −720 m and precision = 460 m), with these high negative values also due to semi‐transparent high clouds. It is worth noting that even in single‐layered cases, we cannot rule out the presence of an optically thick lower layer below (Percentage Opacity = 0.5 can mean a maximum of 7 out of the 14,350‐m Level 1 profiles in the CATS datum were transparent).

For MODIS‐CATS and MISR‐CATS differences, positive values are found as well, primarily for CATS single‐layered clouds. While these positive values comprise 11% of the MODIS‐CATS and 9% of the MISR‐CATS all clouds distributions, it is worth noting that these values extend up to +5 km for MODIS‐CATS, and are mostly due to optically thick top‐layers with mean CTH of 7.8 km; while for MISR‐CATS, these positive differences extend up to about +2.5 km, but are mostly due to optically thick top‐layers with mean CTH of 11.6 km. Positive MISR height bias for high clouds is due to wind‐retrieval bias at those heights (Horváth, 2013); positive MODIS bias for high clouds requires an independent discussion provided in Section 4.5.2.

MISR‐MODIS CTH differences (Figure 7c) do not show striking differences between single‐layered (mode = −80 m, mode *σ* = 670 m) and multi‐layered scenes (mode = −80 m, mode *σ* = 710 m), except in the tail. Overall, MISR and MODIS sense the same cloud to within 1 km of each other nearly 30% of the times – 25% for multi‐layered and 32% of all single‐layered scenes. These scenes constitute the primary peak of the distributions and have a top‐layer mean backscatter of 0.012 (OD∼0.5) and mean altitude of 11.8 km.

**Figure 8:**
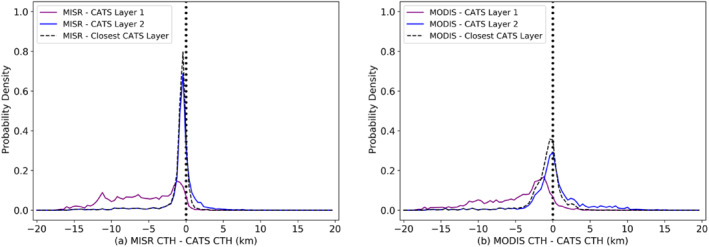
Normalized frequency of occurrence histograms of cloud‐top heights (CTH) differences for double‐layered clouds from Cloud‐Aerosol Transport System (CATS), for each passive sensor—(a) MISR and (b) MODIS—and the first layer (blue lines) and second layer (purple lines) of CATS clouds, respectively, with 100 bins between +20 km and −20 km. The distributions of the difference in cloud top heights from each passive sensor and the closest CATS cloud layer are given by black dashed lines and contain 7,454 collocated data points.

For multi‐layered cases, it is also necessary to quantify which cloud layer the passive sensor is sensitive to; hence, separate histograms of differences between MODIS and MISR CTH and CATS Layer 1 (top layer) and Layer 2 (bottom layer) heights are plotted in Figure 8, for scenes where CATS detected exactly two distinct layers of clouds at least 600 m apart. The difference between MISR or MODIS CTH and the closest CATS layer height is plotted with the thin dashed black line. Figure 8 shows that MISR is highly sensitive to CATS Layer 2 (with mode bias = −400 m and precision = 350 m), seen in the tight overlap of the “MISR—CATS Layer 2” and “MISR ‐ Closest CATS Layer” curves. For scenes where MISR detected CATS Layer 2, the mean and SD of the top‐layer γ (OD) were 0.002 (∼0.08) and 0.0052 (∼0.18), respectively. MISR detection of CATS Layer 1 has mode bias = −1,280 m and precision = 540 m, with a mean and SD top‐layer γ (OD) of 0.01 (∼0.4) and 0.009 (∼0.3), respectively. This supports the existence of a threshold OD necessary for MISR stereo to detect thin cirrus overhanging a textured low cloud, as was suggested in Marchand et al. (2007). One might expect this threshold to be a function of sun‐satellite geometry, texture, and resolution, requiring future investigations using observations and radiative transfer modeling.

Figure 8b shows that MODIS CTH tends to lie between the tops of the two layers as indicated by large negative and positive tails for Layer 1 and Layer 2, respectively, and the closest layer curve is easily distinguishable from the Layer 1 and Layer 2 distributions, unlike the near‐similar profiles for MISR closest layer and Layer 2 distributions, in Figure 8a. For small negative MODIS‐CATS differences, CATS Layer1 is preferred (mode bias = −1,200 m and precision = 1,190 m) as the closest CATS layer (CO_2_‐slicing–negative bias); while for small positive MODIS‐CATS CTH differences, CATS layer 2 (mode bias = 20 m and precision = 850 m) is preferred (BT technique–positive bias). This is consistent with Sections 4.2 and 4.3. When MODIS CTH is more closely associated with the top cloud layer (Layer 1) than Layer 2, the MODIS CTH retrieval is found to lie within the CATS top layer cloud mask 54% of the time, with MODIS CTH being lower than CATS top‐layer base 42% of the time.

### 4.5 CTH Bias and Precision by Instrument

Sections 2, 3, 4 investigated the effects of cloud parameters (top‐layer height and optical depth, and multi‐layering) on the error characteristics of MISR and MODIS CTH retrievals, by assuming CATS CTH to be the truth; these results are summarized in Table 1. However, to constrain our error estimates further, we seek to remove the inherent uncertainty in the collocation process, as well as eliminate the possibility of having multiple layers in a scene. To this end, for the determination of instrument bias and precision, we now restrict ourselves to only single‐layered CATS Level 2 profiles with Percentage Opacity = 1 (i.e., all constituent Level 1 profiles that went into the 5‐km product being opaque), suggesting an absence of broken, multi‐layered clouds and with minimum layer‐integrated OD ∼ 3 (the OD at which a CATS signal is completely attenuated) and where the absolute values of MISR‐CATS and MODIS‐CATS differences are less than 2.5 km (approximately, the largest FWHM from results above). This leaves us with ∼6,000 data points, each for both MISR and MODIS investigation.

**Table 1:** *Moderate Resolution Imaging Spectroradiometer (MODIS) and Multiangle Imaging Spectroradiometer (MISR) Cloud‐Top heights (CTH) Mode Bias and Precision (Rounded to the Nearest Multiple of 10) with Respect to CATS, Summarizing*  2, 3, 4

Parameter of interest	Type of cloud	MODIS		MISR	
		Mode bias (m)	Mode precision (m)	Mode bias (m)	Mode precision (m)
Topmost cloud‐layer height	High (CATS CTH > 5 km)	−1,160	1,080	−540	590
	Low (CATS CTH < 5 km)	40	730	−320	250
Topmost cloud OD (high clouds)	Optically thick (γ > 0.02 sr^−1^)	−280	730	−440	470
	Optically thin (γ < 0.02 sr^−1^)	−1,200	1,020	−680	550
Topmost cloud OD (low clouds)	Optically thick (γ > 0.02 sr^−1^)	500	430	−280	260
	Optically thin (γ < 0.02 sr^−1^)	−440	600	−320	310
Cloud overlap	Single‐layered (high)	−1,160	510	−720	460
	Multi‐layered (highest layer)	−2,380	1,030	N/A^a^	N/A^a^
	Multi‐layered (top layer closest)	−1,200	1,190	−1,280	540
	Multi‐layered (bottom layer closest)	20	850	−400	350

In each row, MISR and MODIS errors are probed by imposing conditions on a cloud “parameter of interest” (e.g., top‐layer height), thus extracting from our dataset a subset of scenes that is representative of a “type of cloud” (e.g., high/low).

^a^ Distribution does not resemble Gaussian.

**Figure 9:**
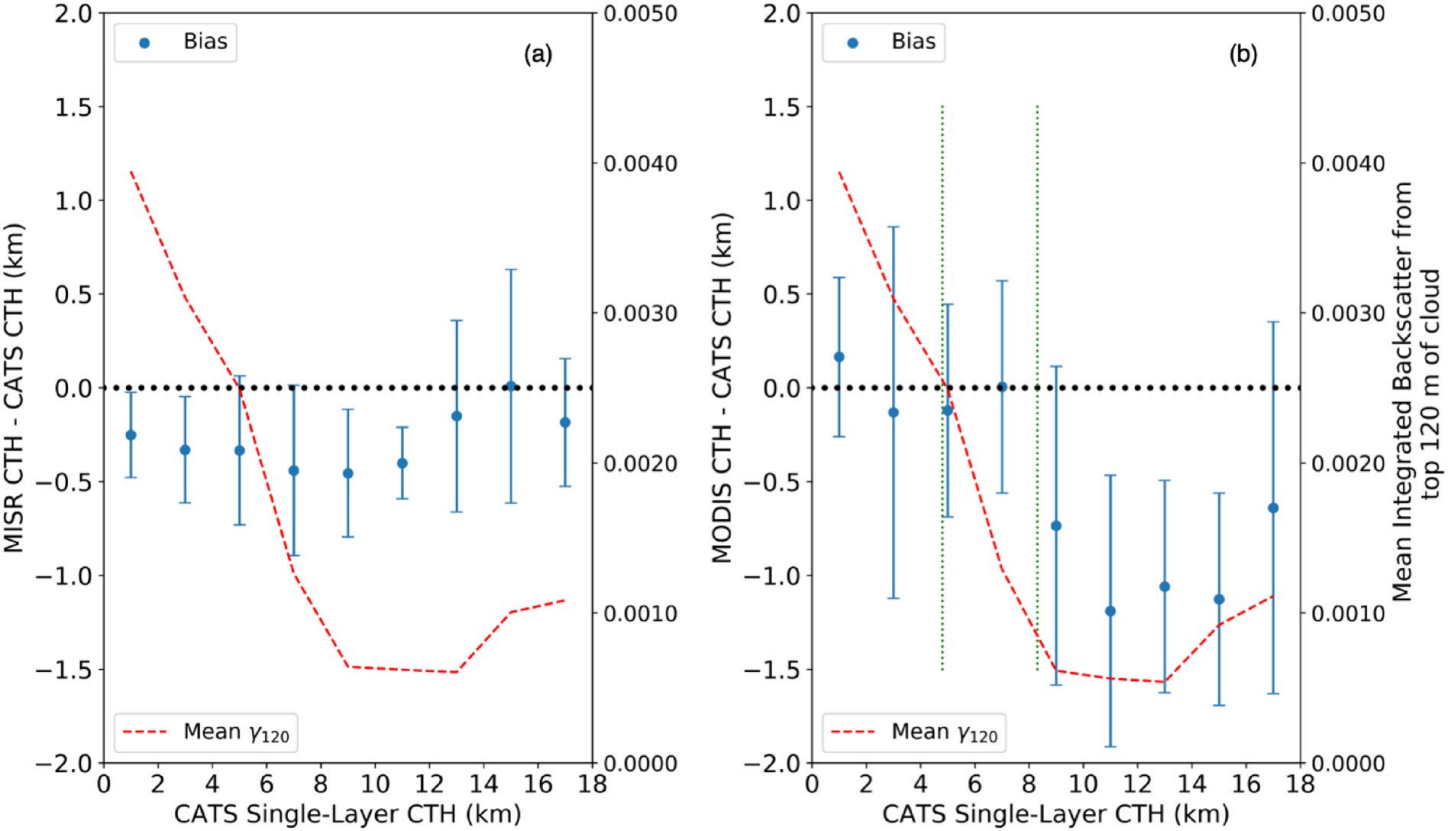
Distribution with altitude of (a) Multiangle Imaging Spectroradiometer (MISR) and (b) Moderate Resolution Imaging Spectroradiometer (MODIS) cloud‐top heights (CTH) mode bias and precision (1σ error‐bars) for cloud‐aerosol transport system (CATS) single‐layered clouds with Percentage Opacity = 1 and an absolute CTH difference ≤2.5 km. The results are binned every 2 km in height (bin centers are odd integers), with mean CATS integrated backscatter for the top 120 m into the cloud (γ_120_ in sr^−1^) shown in red. Green dotted lines in (b) denote the 75th‐percentile CATS CTH for scenes employing IR BT (lower altitude line) and CO_2_‐slicing (upper altitude line), respectively. Each bin has a minimum of 150 samples.

MISR and MODIS bias (offset of the distribution mode from 0) and precision (*σ* from the FWHM approach) are calculated and summarized in Table 2 for all high (CATS CTH > 10 km), mid‐level (10 km > CTH > 5 km), and low‐level (CTH < 5 km) clouds. Moreover, as summarized in Table 1, MISR and MODIS bias and precision exhibit variable dependence on the height and OD of the cloud layer, and to investigate further, Figure 9 shows the distribution of MISR (Figure 9a) and MODIS (Figure 9b) bias and precision (1σ errors‐bars) with altitude (for every 2 km interval) for the same scenes. Each such interval contains a minimum of 150 collocated pixels (∼7–10 independent scenes). The mean CATS integrated backscatter from cloud top to 120 m below cloud top, γ_120_, is also shown for each bin. It is readily apparent from both Table 2 and Figure 9 that MISR exhibits lower bias and greater precision than MODIS CTH. MISR precision is found to be remarkably consistent with cloud altitude, varying between 200 and 550 m, whereas MODIS precision generally deteriorates from ∼500 m for the lowest to ∼900 m for the highest clouds. A detailed discussion of the error budget for MISR and MODIS CTH is provided in the next two sub‐sections.

**Table 2:** Multiangle Imaging Spectroradiometer (MISR) and Moderate Resolution Imaging Spectroradiometer (MODIS) Mode bias and Precision (Rounded to Nearest Multiple of 10) for all, High, Mid‐level, and Low Clouds, as Seen by Cloud‐Aerosol Transport System (CATS), With Absolute Cloud‐Top Heights (CTH) Difference With Respect to CATS ≤ 2.5 km and CATS Percentage Opacity = 1

Instrument	Overall		High (CATS CTH > 10 km)		Mid‐level (10 km > CTH > 5 km)		Low (CATS CTH < 5 km)	
	Mode bias (m)	Mode precision (m)	Mode bias (m)	Mode precision (m)	Mode bias (m)	Mode precision (m)	Mode bias (m)	Mode precision (m)
MISR	−280	370	−300	400	−370	400	−240	300
MODIS	−540	690	−950	740	−350	690	60	660

#### 4.5.1 MISR CTH Errors

The MISR bias reported in Table 2 arises from three principal sources–bias in co‐registration of oblique radiances with nadir, wind‐retrieval bias, and a stereo‐opacity bias (retrieval of stereo height at a depth into the cloud due to low extinction near the top). We assume CATS CTH to be unbiased. Sources of random error that determine the overall MISR CTH precision include geo‐registration errors of MISR imagery, correspondence errors of conjugate cloud features in MISR imagery, random wind‐retrieval errors, and random sub‐pixel CTH variability due to geo‐collocations (∼300 m from Section 3). We assume the random error in CATS CTH to be the result of equal probability of successful and failed detection over the depth of one range‐gate: thus, contributing a random error of 30 m. Globally, MISR image geo‐registration error is estimated to be 0.05 ± 0.25 pixels, which translates to height errors of about 30 ± 140 m (Davies et al., 2007; Jovanovic et al., 2007).

Wind‐retrieval errors also propagate to height errors, although these contributions have been reduced from the TC_STEREO to TC_CLOUD product (Horváth, 2013; Lonitz & Horváth, 2011; Mueller et al., 2017). Comparison of MISR near‐surface heights to ground targets allows for the evaluation of CTH errors due to the combined effects of registration, correspondence, and DEM errors, as done in Horváth (2013). We repeated their analysis using MISR data between 50°N and 50°S, finding the mode in height to be = −40 m and mode *σ* = 170 m, using the FWHM approach. These values are similar to the values (mean height error = −31 m, RMS error = 171 m) reported in Horváth (2013).

If the bias was not a function of wind speed, then we would conclude for the overall cloud samples used in Table 2 that the stereo‐opacity bias is −280 m + 40 m = −240 m (−260 for high clouds; −200 m for low clouds). However, based on the analysis of Horváth (2013), wind errors do vary with altitude based on comparisons with geostationary wind data, with an along‐track wind bias of ∼1 m s^−1^ for low clouds and ∼1.2 m s^−1^ for high clouds, respectively (cross‐track winds are essentially unbiased). Using ∼90 m height error for each 1 m s^−1^ error in along‐track wind‐speed (Mueller et al., 2017, Table 1), these wind‐errors translate to CTH errors of ∼90 m for low clouds and ∼110 m for high clouds. Thus, we deduce that the stereo‐opacity bias is ∼ −110 m (= −200 m + 90 m) and ∼ −150 m (= −260 + 110 m) for low and high clouds, respectively. As the very name suggests, one might expect the stereo‐opacity bias to be strongly dependent on the extinction in the ∼100 m region below the cloud‐top. Here, we use the layer‐integrated backscatter in the top 120 m of clouds (γ_120_) to segregate very thin cloud tops (γ_120_ < 0.005 sr^−1^ or OD_120_ < 0.18, over the depth of the top 120m) from the comparatively thicker cloud tops (γ_120_ > 0.005 sr^−1^). For the very thin clouds, the stereo‐opacity bias is found to vary between −130 m and −180 m in high and low clouds, respectively, whereas for larger γ_120_, the range of estimated stereo‐opacity ranges between −70 and −100 m. Thus, we can nominally estimate the full range of stereo‐opacity bias (subject to estimated wind‐correction bias) to be −60 to −200 m for low clouds and between −100 and −260 m for high clouds. The difference in stereo‐opacity bias between high and low clouds is likely due to lower clouds having larger γ_120_ (i.e., greater extinction coefficients in the upper parts of the cloud; Figure 9a). Of course, there is uncertainty in the wind‐speed bias given that they are derived from comparisons with geostationary AMVs. Using a conservative estimate of ±0.5 m s^−1^ error in wind‐speed bias leads to a ±45 m shift in wind‐correction and stereo‐opacity CTH biases.

For the MISR CTH precision budget, we noted earlier that the MISR CTH co‐registration/correspondence/DEM precision = 170 m and that the maximum CTH geolocation precision of our method = 300 m. But, since we are dealing with Percentage Opacity = 1 in this section, we expect the geolocation‐related variations in heights to be much smaller here, and our overall observed precision of 370 m may be almost entirely dictated by the precision of MISR stereo. Here, it should be noted that our observed precision is about twice as good as was reported in both Horváth (2013) and Mueller et al. (2017) and is most likely due to the highly precise CTH that a lidar is able to offer (taken here as 30 m) compared to the IR AMV heights used in those studies. Assuming geolocation‐related height error = 0 m, and the overall MISR precision from Table 2 to be 373 m, the MISR wind‐height precision =  $\sqrt{370^{2}-170^{2}-30^{2}}$ = 330 m (360 and 250 m for high and low clouds, respectively). Using the 90 m (m s^−1^)^−1^ wind‐height error proportionality again, we get an overall MISR wind speed precision of 3.7 m s^−1^ (4.0 and 2.8 m s^−1^ for high and low clouds, respectively). Our MISR wind speed precision estimates backed out through MISR‐CATS comparison are remarkably close (to within 5%–10%) to those determined by both Horváth (2013) and Mueller et al. (2017), thus providing closure. This result also implies that the MISR operational quality assurance procedures, most notably the required agreement (and subsequent averaging) of forward and aft‐derived height estimates, are filtering and improving the accuracy of raw stereo retrievals to an extent that mitigates the difficulty of obtaining heights from highly dynamic or poorly textured clouds.

#### 4.5.2 MODIS CTH Errors

A similar accounting of MODIS CTH bias and precision is not strictly possible as MODIS uses *a priori* assumptions and ancillary data to retrieve CTHs. Its errors covary with the departures from these assumptions and deviations from reality in the ancillary data. The CTH uncertainties in IR sensors have been historically reported as CTP errors (Menzel et al., 2015; Wielicki & Coakley, 1981), although in recent literature (Baum et al., 2012; Holz et al., 2008), CTH errors have been quantified by comparing low‐level and single‐layered clouds against lidar. For example, from Figure 12 of Baum et al. (2012), we can estimate the bias and precision (FWHM method as above) to be −1,100 and 930 m, respectively, for single‐layered cirrus, and a bias and precision of 200 and 550 m for low clouds. The corresponding values of bias and precision for high and low clouds in Table 2 are quite similar, even though we define high and low clouds differently than that study. The MODIS bias (Table 2) seems to be largely due to high clouds, which goes back to systematic bias in the CO_2_‐slicing technique, which employs an infinitesimally thin cloud assumption. In these high cloud samples (optically thick cirrus), the negative bias presumably arises because optically thick cirrus also tends to be geometrically thick, leading to CO_2_‐slicing underestimating CTH. Again, owing to the choice of Percentage Opacity = 1, MODIS precision is assumed to be mostly unaffected by collocation errors and originates from the forward modeling.

Systematic CTH overestimation by MODIS for low clouds and underestimation for semi‐transparent high clouds is due to the retrieval techniques it employs and cannot be explained by just top‐layer height, OD and overlap. For MODIS, the low and high cloud distinction used here nearly coincides with the 75th percentile heights (green‐dashed lines in Figure 9b) where IR BT and CO_2_‐slicing techniques are applied, whereas mid‐level clouds employ both. As a result, the bias and precision of the two techniques can be roughly estimated by MODIS bias and precision for high and low clouds (Table 2).

**Figure 10:**
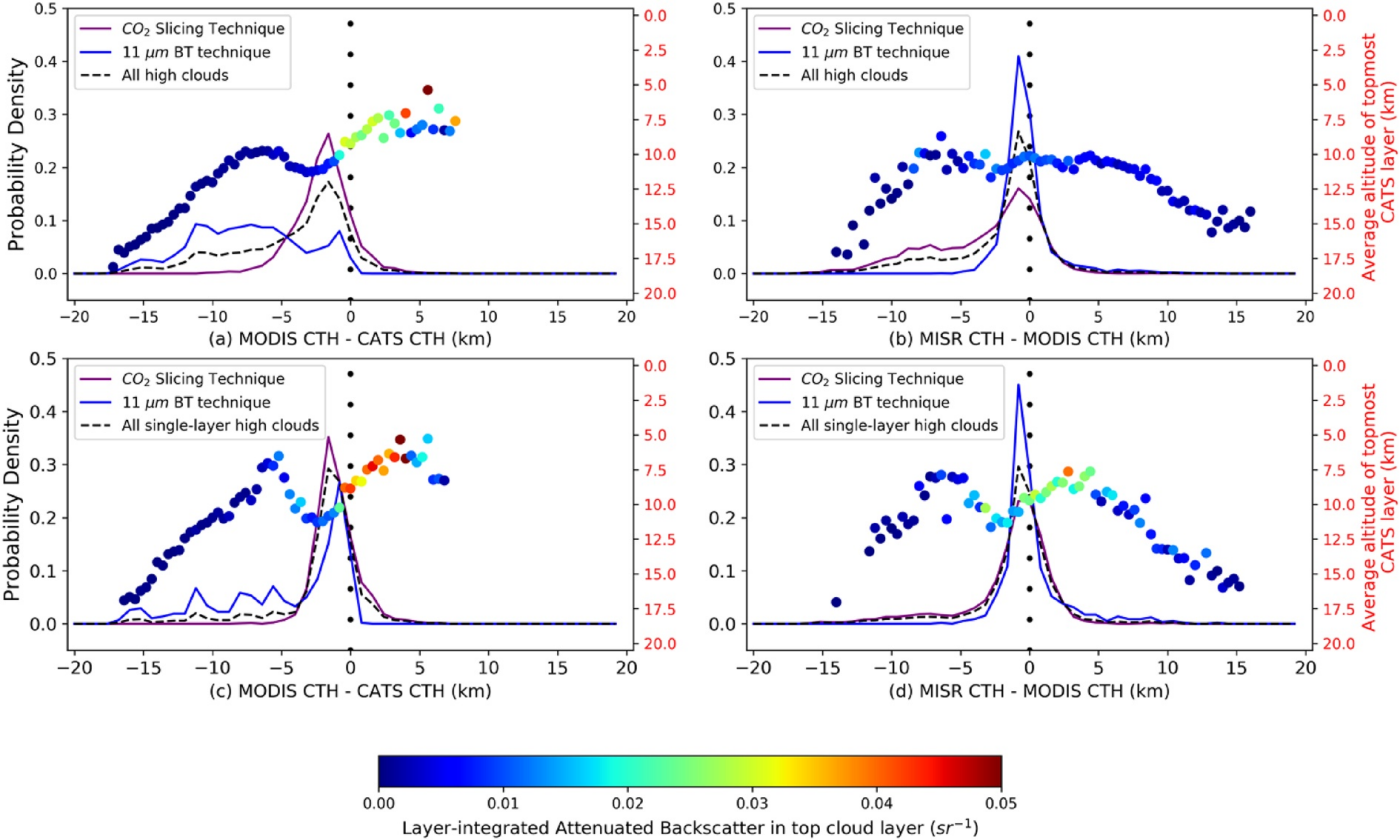
Normalized frequency of occurrence histograms of cloud‐top heights (CTH) differences for CATS high (CTH > 5 km) clouds (upper panel) and cloud‐aerosol transport system (CATS) single‐layered high clouds (lower panel). CO_2_‐slicing retrievals are in purple and 11‐μm brightness temperature retrievals are in blue. The mean of the CATS CTH for all top layers in a histogram bin is represented by a large colored dot and is associated with the red *y*‐axis to the right of each panel. The color of the dot itself represents the CATS layer‐integrated backscatter (γ) for the topmost cloud layer.

To investigate the MODIS CTH bias and precision for the two CTH techniques, Figure 10 presents histograms for MODIS‐CATS (Figures 10a and 10c) and MISR‐MODIS (Figures 10b and 10d) CTH differences for all CATS high (CTH > 5 km) clouds (top panel) and single‐layered high clouds (bottom panel). Only high cloud retrievals are chosen to focus on scenes where CO_2_‐slicing is preferred, but IR BT is still possible. A simple pressure‐based distinction is not applicable as CO_2_‐slicing is only reserved for ice clouds. Aqua‐MODIS phase flag is understood to accurately determine ice phase 65%–80% of the time globally, through inter‐comparisons with CLOUDSAT/CALIPSO data, with >90% agreement for multiple surface types for single‐phase clouds (Marchant et al., 2016; Platnick et al., 2017). For the data plotted in Figure 10, 57% of all collocated high clouds and 70% of single‐layered high clouds were retrieved using CO_2_‐slicing, in keeping with the improvements of Collection six MOD06 updates aimed at increasing frequency of CO_2_‐slicing retrievals (Baum et al., 2012). In both high and single‐layered high clouds, the smallest differences are associated with CO_2_‐slicing for MODIS‐CATS and with IR BT technique for the MISR‐MODIS. This discrepancy is because CO_2_‐slicing is more sensitive to optically thin high clouds than MISR and has a mean CTH closer to mean CATS CTH in most cases (especially, single‐layered high clouds), as shown in Figure 5. However, due to reasons explained earlier, MODIS often detects mid‐tropospheric CTH about 3–5 km above MISR CTH. Large MODIS‐lidar differences occur for IR BT technique, as noted in previous studies (Holz et al., 2008; Naud et al., 2004), for semi‐transparent high clouds (OD < 1), where MODIS opts for the IR BT technique over the more precise CO_2_‐slicing. The mean MODIS CTH error associated with the application of the IR BT technique is found to be −5.8 km overall for scenes with CATS CTH > 5 km, with CATS mean top‐layer backscatter less than 0.02 sr^−1^ and mean top‐layer height greater than 10 km. For CATS single‐layered high clouds, mean CTH error from the application of IR BT is −2.3 km.

For MODIS‐CATS difference (Figures 10a and 10c), positive values in the overall distributions (black dashed lines) are associated with optically thick, single‐layered high clouds (as in Figure 6a) with top‐layer backscatter greater than 0.01 sr^−1^ and CTH between 5 and 10 km. This is clearly associated with CO_2_‐slicing and not IR BT and is difficult to explain based on the information at hand. CO_2_‐slicing is subject to many sources of errors—instrument noise, uncertainties in calculating clear‐sky radiances, assumption of constant emissivity in band‐pairs used to calculate CTP and deviations from constant lapse rates. Apart from these, there may be two more sources of error for the present data. First, CATS, unlike the CALIOP lidar used in Holz et al. (2008), employs a single horizontal resolution (5 km) for layer‐detection and is known to miss extremely tenuous cirrus layers during daytime (Rajapakshe et al., 2017). As a result, it might be possible that MODIS 1‐km CTH can detect sub‐5 km, thin higher cirrus that CATS might miss. Second, a problem endemic to Terra MODIS, but not with Aqua‐MODIS used in Holz et al. (2008), is that one of the bands used in the CO_2_‐slicing—Band 34 (13.6 μm)—remains unused due to severe noise, effectively reducing the algorithm to just 14.2/13.9 and 13.9/13.3 μm ratios (most sensitive to pressure regimes of 100–450 hPa and 550–650 hPa, respectively), instead of the full suite of options (Menzel et al., 2008). Hence, in this analysis alone, 73.1% of all CO_2_‐slicing retrievals for high clouds and 78% for single‐layered high clouds were from the 14.2/13.9 μm band‐pair, while the remainder came from 13.9/13.3 μm band‐pair. The important 35/33 (13.9/13.6 μm) band pair, most sensitive to mid‐level clouds and cloud edges (Menzel et al., 2015), is missing and is a possible reason for overestimation of mid‐level CTH.

## 5 Conclusions

Terra is our longest running single‐platform mission with a stable ECT for CTH, now spanning more than 2 decades. Its long record from a stable orbit makes it valuable in climate research and in data assimilation in reanalysis products. Of course, its scientific application requires well characterized errors in the public geophysical products produced by the Terra mission. Here, we have used the ISS CATS lidar to quantify the error characteristics of MODIS and MISR CTHs from Terra, producing the first evaluation of these errors from space‐based lidar, on a semi‐global domain between ±50° latitudes. Ample collocated (<1 km) and concurrent (<5 min) MODIS, MISR, and CATS samples were retrieved during the CATS 2015–2017 period for robust statistics. While CATS top‐layer CTH is taken as truth in our analysis, the CATS‐detected lower‐level cloud tops underlying thin upper‐level clouds were also used to examine MODIS and MISR CTH error characteristics—an approach that proved to be central in our understanding of MISR and MODIS CTH. Generally, we find that MISR and MODIS CTH errors are larger in the tropical regions and smaller in the midlatitudes and are strong functions of cloud type, defined by cloud height, optical depth, and multi‐layering, as summarized in Tables 1 and 2. Although the sampling of the midlatitudes is more frequent than that of the tropics in our collocated dataset, the conservatively estimated global standard error for our samples is considerably low (≤5% of 1σ, for all cases reported).

For CATS CTH <5 km (single and multi‐layered), MISR and MODIS CTH mode biases and precisions (mode bias ± precision) are −320 ± 250 m and 40 ± 720 m, respectively. MISR CTH bias changes little with optical depth (Figure 6), but a reduction of MISR CTH mode bias to −240 m for unbroken, single‐layered, and opaque low clouds is observed (Table 2). In contrast, MODIS CTH bias for low clouds (hence, IR BT technique) is highly dependent on optical depth with a mode bias of −440 m for thin clouds (*γ* < 0.02 sr^−1^ or OD < 0.8) and of +500 m for thick clouds (*γ* > 0.02 sr^−1^) (Table 1). This dichotomy occurs because for optically thinner (more transmissive) clouds, the IR BT technique senses a thermal signature of the warmer surface, whereas, for high OD (more emissive) clouds, there is presumably greater lapse rate deviation from the climatology used in Collection six MOD06 product. When considering the subset of unbroken, single‐layered, and opaque low clouds, MODIS CTH mode bias is +60 m (Table 2), with the positive bias for more emissive clouds dominating, as low clouds tend to be thicker on average in our dataset.

For CATS CTH >5 km (single and multi‐layered), MISR and MODIS CTH mode biases are −540 ± 590 m and −1,160 ± 1,080 m, respectively. For both MISR and MODIS, high cloud biases do tend to vary with optical depth. MODIS CTH mode bias is −1,200 m for thin high clouds (*γ* < 0.02 sr^−1^) and −280 m for thick clouds (*γ* > 0.02 sr^−1^). Low opacity near cloud‐top in geometrically thick clouds leads to underestimation of MODIS CTH as CO_2_‐slicing technique assumes an infinitesimally thin single‐layered cloud solution. Similarly, the MISR CTH mode bias is −680 m for high clouds with *γ* < 0.02 sr^−1^ and −440 m for those with *γ* > 0.02 sr^−1^, suggesting the presence of a stereo‐opacity bias–the depth into the cloud in which spatial contrast is established in the emerging radiation field. This study provides the first assessment of the MISR stereo‐opacity bias, estimated here to range between −60 and −260 m for clouds sampled in this study, subject to the accuracy of known MISR wind‐speed errors. It is larger for higher altitude clouds owing to the optically thinner nature of cloud tops for higher altitude clouds.

For CATS‐retrieved multi‐layered clouds, which are often thin cirrus (*γ* ≤ 0.02 sr^−1^) overlying thicker clouds, CTH comparisons are more complicated. Both passive sensors severely underestimate top‐layer CTH, MISR by −1,280 ± 540 m and MODIS by −1,200 ± 1,190 m. These large biases necessitate us to adopt a “closest layer” approach (i.e., comparing passive‐sensor CTH to closest CATS layer height). For two‐layered cases, MISR is found to be sensitive to the lower cloud layer, with MISR CTH errors for this lower layer being −400 ± 350 m. This is almost identical to MISR single‐layered low cloud bias and precision, suggesting that MISR low CTH accuracy is independent of the presence of a high, thin cirrus. The mean top‐layer OD when MISR detects the higher layer is found to be 0.4 ± 0.3, agreeing with the result from Marchand et al. (2007). This is indicative of an opacity threshold for stereo detection, a parameter which would presumably be a function of sun‐satellite geometry and spatial contrast. MODIS underestimates top‐layer CTH by greater than 1 km due to the CO_2_‐slicing technique converging at a higher‐pressure solution, when an optically thin (OD < 0.8) cloud is present. As a result, MODIS produces more midlevel CTH than MISR and MISR‐MODIS CTH differences have generally low absolute values.

Optically thick, single‐layered, unbroken clouds allow us to neglect random collocation errors (∼300 m) for a complete error budget analysis for MISR stereo. Unlike MODIS, the MISR CTH error budget is self‐contained since it does not rely on external ancillary products. MISR underestimates CTH for these clouds by −280 ± 370 m. Contributors to the bias are estimated as: (a) bias in imagery co‐registration and feature correspondence (∼−40 m), (b) MISR stereo‐opacity bias (−110 to −150 m, dependent on cloud altitude) and (c) MISR wind‐correction bias (−60 to −260 m, also dependent on altitude). The estimated wind‐correction and stereo‐opacity bias is, however, subject to the accuracy of previously reported estimates of wind‐speed bias. Random errors in this dataset are largely due to wind‐driven errors (330 m for all samples, 250 m for low and 360 m for high clouds). Based on our estimated wind‐height precision, we were able to provide an independent estimate of MISR wind‐speed precision of 3.7 m s^−1^ (2.8 and 4.0 m s^−1^ for low and high clouds, respectively). These values are quite similar to the findings of Horváth (2013) and Mueller et al. (2017). Thus, we conclude that we have essentially achieved closure on the MISR CTH error budget.

Similarly, MODIS underestimates CTH by −540 ± 690 m for these optically thick, single‐layered, and unbroken clouds in our dataset. While it is difficult to exactly quantify, the largest contributor to MODIS CTH bias is the CO_2_‐slicing underestimation for geometrically thick cirrus. MODIS CTH random errors are due to inherent uncertainties in the forward model and reliance on external ancillary datasets. Since CO_2_‐slicing is best suited for thin cirrus, application of IR BT for high clouds (when CO_2_‐slicing does not converge to solution) can still lead to erroneous results, as discussed in Holz et al., (2008). However, compared to Collection five, improvements of Collection six low‐cloud CTH from the marine boundary‐layer correction, as well in high‐cloud retrievals from adopting CO_2_‐slicing technique more frequently (Baum et al., 2012), have indeed led to a substantial reduction in errors in MODIS CTHs.

Our findings allow us to make some reasonable suggestions concerning the use of these data. While it seems perfectly reasonable to always choose MISR CTH over MODIS CTH given their uncertainties summarized above, caution is required. Under multi‐layered conditions in which thin higher clouds are overlapping lower textured clouds, MISR will be reporting the CTH of the lower textured cloud. And under such conditions, MODIS CTH will carry a large uncertainty as described above. These conditions, however, may be flagged by either applying a threshold to MODIS‐MISR CTH differences or applying the MODIS multi‐layered flag (Wind et al., 2010). In such cases, fusing MISR and MODIS provides pixel‐level CTH of two distinct cloud layers, each with uncertainties as reported above; hence, it increases our knowledge of cloud cover distributions over either single instrument alone. For single layer clouds, it may be tempting to simply average the MODIS and MISR CTHs. But the precision of this averaged CTH would be degraded relative to that of MISR since the precision of the MODIS CTH is greater than that from MISR by a factor of 3^1/2^. There is also the issue of detection, which we did not address here, whereby some locations may report CTH from one instrument but not the other. Here, it appears reasonable to choose CTHs that are reported in order to increase the representativeness of clouds over what any single instrument alone can provide, keeping in mind that the uncertainties of these CTH values are not ascertained here (we only examined CTH where both MISR and MODIS have valid CTH retrievals for the same location). Finally, we point out that our results are strictly valid at the near‐nadir view of CATS. We expect them to apply over the MISR swath for MISR CTH since their errors exhibit little dependence in the cross‐track direction in the version used here (Mueller et al., 2013). We are less certain of this for MODIS given that MODIS CTH exhibit cross‐track variability in the mean (Maddux et al., 2010).

Our findings also point to recommendations for future satellite architecture designs that have CTH as a target product, such as the Aerosol and Cloud, Convection, and Precipitation (ACCP) mission called out in National Academies of Sciences, Engineering, and Medicine (2018). As each of these sensors (lidar, IR, multi‐view) occupies a niche that cannot be replaced by the others alone, these sensors on a single‐orbit taking observations of the same physical reality can improve the short‐comings of each by creating fused datasets that complement each other and provide greater insight to CTH variability than any of these sensors operating alone. Also, our analysis and closure of the MISR CTH error budget has several implications for future stereo‐enabled technological designs. Since the largest contributor to the error budget is wind‐driven errors, removing this error can be achieved by flying two (or more) multi‐view imaging systems in close proximity and in close formation. This would allow for the same scene to be viewed at the same time, hence removing wind‐driven errors. Improving resolution would also improve the precision in the stereo CTH (an instrument resolution of ∼100 m would contribute ∼60 m to the precision budget, assuming MISR viewing geometry). We recommend that detailed 3D radiative transfer modeling would be undertaken to fully understand the nature of the remaining stereo‐opacity bias–how it varies with sun‐satellite geometry and cloud micro‐ and macro‐physical properties.

## Data Availability

The MODIS data were obtained through the Level 1 and Atmosphere Archive and Distribution System of NASA Goddard Space Flight Center (https://ladsweb.modaps.eosdis.nasa.gov/archive/allData/61/). The CATS data were downloaded from the NASA Langley Research Center’s ASDC DAAC (https://opendap.larc.nasa.gov/opendap/CATS/). The MISR data were downloaded from NASA Langley Research Center Atmospheric Sciences Data Center (https://opendap.larc.nasa.gov/opendap/MISR/MIL2TCSP.001/).
